# Investigating the Roles of Active Site Residues in *Mycobacterium tuberculosis* Indole-3-glycerol Phosphate Synthase, a Potential Target for Antitubercular Agents

**DOI:** 10.1021/acsbiomedchemau.3c00029

**Published:** 2023-07-26

**Authors:** David W. Konas, Sarah Cho, Oshane D. Thomas, Maryum M. Bhatti, Katherine Leon Hernandez, Cinthya Moran, Hedda Booter, Thomas Candela, Joseph Lacap, Paige McFadden, Savannah van den Berg, Alyssa M. Welter, Ashley Peralta, Cheryl A. Janson, Jaclyn Catalano, Nina M. Goodey

**Affiliations:** Department of Chemistry and Biochemistry, Montclair State University 1 Normal Avenue, Montclair, New Jersey 07043, United States

**Keywords:** indole-3-glycerol phosphate synthase, IGPS, *Mycobacterium tuberculosis*, tryptophan biosynthesis, enzyme catalysis

## Abstract

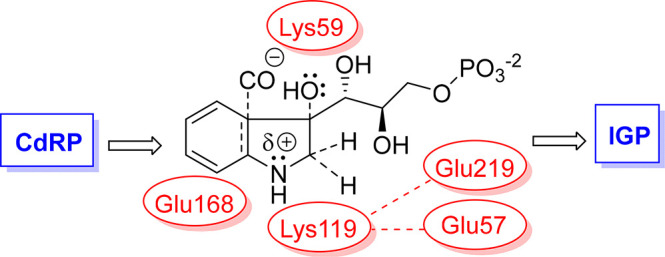

*Mycobacterium tuberculosis* drug resistance is emerging and new drug targets are needed. Tryptophan biosynthesis is necessary for *M. tuberculosis* replication and virulence. Indole-3-glycerol phosphate synthase (IGPS) catalyzes a step in *M. tuberculosis* tryptophan biosynthesis and has been suggested as a potential anti-infective target, but our understanding of this enzyme is limited. To aid in inhibitor design and gain a greater mechanistic picture of this enzyme, there is a need to understand the roles of active site amino acids in ligand binding and catalysis. In this work, we explored the roles of conserved active site amino acids Glu57, Lys59, Lys119, Glu168, and Glu219. Mutation of each to Ala results in loss of all detectable activity. The Glu57Gln, Lys59Arg, Lys119Arg, Glu168Gln, and Glu219Asp mutations result in large activity losses, while Glu219Gln has enhanced activity. Analysis of the enzymatic data yields the following main conclusions: (A) Lys119 is the likely catalytic acid in the CdRP ring closure step. (B) Glu168 stabilizes a charged reaction intermediate and may also be the catalytic base. (C) Glu57, Glu219, and Lys119 form a closely arranged triad in which Glu57 and Glu219 modulate the p*K*_a_ of Lys119, and thus overall activity. This increased understanding of inter- and intramolecular interactions and demonstration of the highly coordinated nature of the *M. tuberculosis* IGPS active site provide new mechanistic information and guidance for future work with this potential new drug target.

## 1 Introduction

Tuberculosis (TB), caused by *Mycobacterium tuberculosis* (*Mt*),^1^ is an ancient infectious disease^2^ that is still a serious health issue today due to waning effectiveness of current therapeutics. Efficacy of first-line antibiotics rifampicin and isoniazid is reduced in multidrug resistant strains,^3^ and second-line drugs are also less potent now against extensively drug-resistant strains.^4−6^ TB/HIV co-infections are an issue in developing countries.^7,8^ Even when the drugs are effective, successful outcomes are complicated by lack of patient compliance since full treatments involve relatively long time frames from 6 to 24 months.^1^

Consideration of *Mt* metabolism in the context of the host immune response led to the identification of a much-needed new anti-TB strategy. Both latent *Mt* infection and active disease are associated with decreased serum and plasma tryptophan (Trp) levels.^9^*Mt* living in macrophages should be able to access both macrophage tryptophan and its own biosynthesized tryptophan, but the human CD4 T cell response decreases macrophage tryptophan concentrations via the action of cytokine IFN-γ and indoleamine-2,3-dioxygenase (IDO).^10^ In this Trp-deficient environment, *Mt* likely relies on its own Trp production for optimal growth and survival.^11,12^ In fact, Trp synthesis genes are overexpressed during immune stress, so inhibition of this pathway may assist the immune system in fighting *Mt* infections.^13^ Smith et al. showed that genes coding for tryptophan biosynthesis pathway enzymes are essential for *Mt* virulence.^14^ As a result, recent work has explored strategies to inhibit tryptophan synthase, an enzyme downstream from indole-3-glycerol phosphate synthase (IGPS) in the *Mt* tryptophan biosynthesis pathway.^15,16^

**Figure 1:**
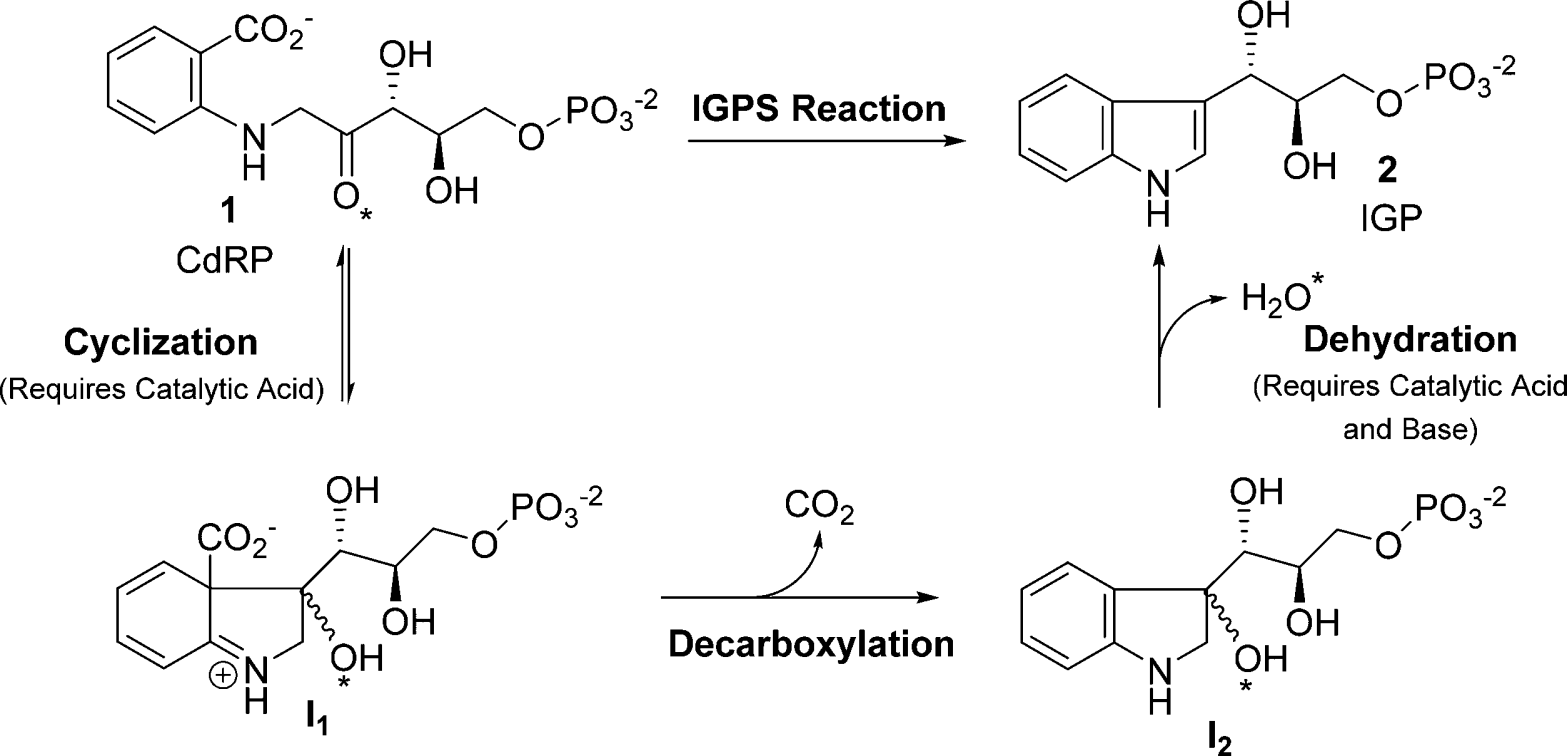
Overall reaction and proposed intermediates for the *Mt*IGPS reaction. Steps where a catalytic acid or base is required (cyclization and dehydration steps) are indicated (see parentheses). Lys119 is proposed to act as a catalytic acid in the cyclization step. The star (*) identifies the carbonyl oxygen in CdRP, which leaves as H_2_O in the dehydration step.

Indole-3-glycerol phosphate synthase (IGPS) catalyzes the fourth step of Trp synthesis in bacteria. Sassetti et al. showed that the gene encoding *Mt*IGPS is essential for growth of the pathogen *in vitro*.^11^ An *Mt*IGPS inhibitor, ATB107, inhibited the growth of drug-sensitive and drug-resistant strains.^17^*Mt*IGPS catalyzes the conversion of 1-(*o*-carboxyphenylamino)-1-deoxyribulose-5-phosphate (CdRP, **1**) to indole-3-glycerol phosphate (IGP, **2**) accompanied by the release of CO_2_ and H_2_O (Figure 1). The aromatic rings of the substrate and product occupy different binding sites and conformational motions must take place throughout the catalytic process.^18^ The working IGPS mechanism hypothesis is based on a proposal by Parry^19^ and involves two intermediates. Enamine-like cyclization yields the first (**I**_**1**_) followed by decarboxylation/rearomatization to form the second (**I**_**2**_). Finally, dehydration of **I**_**2**_ yields the product IGP. The overall reaction is assumed to be irreversible due to the release of CO_2_ and formation of the 10-π aromatic indole. At least one active site acid is required to facilitate two different protonations of the oxygen (O*, Figure 1) accompanying the cyclization and dehydration steps, and an active site base is required for deprotonation accompanying the dehydration.

Both experimental and computational data for other IGPS enzymes indicate that a conserved lysine corresponding to Lys119 in *Mt*IGPS functions as a catalytic acid and likely mediates the ring closure leading to formation of the first intermediate (IGPS to **I**_**1**_, Figure 1).^20−23^ Lys59 on the β1α1 loop in *Mt*IGPS has been proposed as the proton source in the subsequent dehydration step (**I**_**2**_ to IGP; Figure 1). For example, in their work with *Ss*IGPS, Zaccardi and co-workers implicated the corresponding Lys53 in that protein as the general acid in this step (Figure S13).^21^ Three different conserved glutamate residues have been proposed to serve as the general base in the IGPS dehydration step (**I**_**2**_ → IGP, Figure 1). Hennig et al. proposed it is Glu159 in *Ss*IGPS (corresponding to Glu168 in *Mt*IGPS) and Czekster et al. also suggested it is Glu168 in *Mt*IGPS (see amino acid alignment in Figure S13).^23,24^ Mazumder-Shivakumar and Bruice suggested that it is Glu210 in *Ss*IGPS (corresponding to Glu219 in *Mt*IGPS) based on molecular dynamics simulations.^20^ Finally, Zaccardi et al. reported data consistent with Glu51 as the *Ss*IGPS catalytic base (corresponding to Glu57 in *Mt*IGPS).^21^ Based on previously published data, none of these three can be definitively identified or ruled out as the IGPS dehydration base. Zaccardi et al. proposed that the residue corresponding to Glu219 is Glu210 in *Ss*IGPS and it is involved in substrate capture while Mazumder-Shivakumar and Bruice proposed Glu210 in *Ss*IGPS as the catalytic base in the dehydration step (Figures 1 and S13).^20,21^

Some work has been done to identify these key acid/base residues in IGPSs from other organisms;^21,22,25^ however, existing mutagenesis data from *Mt*IGPS are quite limited despite an urgent need to better understand this enzyme target from the highly pathogenic *Mt*. There is only one existing paper containing any mutagenesis data for the *Mt*IGPS enzyme.^17^ This work by Shen and co-workers reports only *K*_M_ values for a small set of alanine mutations, and conclusions from the data are limited to information on substrate binding. In this work, we greatly expand the body of mutagenesis data for *Mt*IGPS by examining numerous active site mutations at positions Glu57, Lys59, Lys119, Glu168, and Glu219, a collection including some that have only been explored in the corresponding positions in other IGPS homologues and others that have not yet been studied in any IGPS. Steady-state kinetics and rate versus pH relationships were examined for wild-type and mutants, and the data provide insights into the importance and potential catalytic roles of these residues. We also used computer modeling to dock the substrate CdRP into an *Mt*IGPS active site crystal structure to predict interactions between the substrate and active site residues. *Mt*IGPS represents a potential target for new anti-TB agents and understanding its substrate binding and catalytic mechanism will facilitate design of more specific inhibitors and probes of this interesting indole-forming enzyme.^26^

## 2 Experimental Procedures

### 2.1 Site-Directed Mutagenesis

The wild-type *MtIGPS* gene with an N-terminal 6-His tag in the pET30a vector was purchased from Genewiz. *Mt*IGPS genes mutated at Glu57, Lys59, Glu168, Glu219, or Lys119 were also purchased or prepared using Quick Change mutagenesis^27^ and confirmed by sequencing.

### 2.2 Expression, Purification, and Characterization of *Mt*IGPS Enzymes

The pET30a vector containing the trpC coding sequence for wild-type or a mutant was transformed into *E*. *coli* BL21 LOBSTR cells.^28^ A previously reported expression and purification protocol was followed with modifications.^29^ Transformants were grown in LB with 50 μg/mL kanamycin at 37 °C to *A*_600_ 0.5–0.6. Isopropyl-d-thiogalactopyranoside (IPTG) was added to 1.0 mM, and the cells were incubated 16–18 h at 25 °C with shaking (225 rpm). The cells were harvested, resuspended in 15 mL of equilibration buffer (1× PBS, 10 mM imidazole, pH 8.0), and sonicated. The lysate was centrifuged and loaded onto a Ni-NTA His affinity resin. The resin was washed with 1× PBS, 40 mM imidazole, and 100 mM NaCl, pH 8.0. *Mt*IGPS was eluted with 1× PBS, 250 mM imidazole, pH 8.0, at 4 °C with yields of 0.5–7.0 mg/L culture. Purified *Mt*IGPS was dialyzed against 5 mM Tris, 20 mM NaCl, and 2 mM DTT (pH 7.9) and stored at −80 °C with 10% glycerol for cryoprotection. Protein purity was confirmed by SDS-PAGE. Protein concentration was determined at 280 nm (ε = 4595 M^–1^ cm^–1^). CD spectra were collected for wild-type and *Mt*IGPS mutants in 2.5 mM Tris, 10 mM NaCl, pH 7.5, using an Applied Photophysics Chircascan (bandwidth 0.5 nm, wavelength range 190–300 nm, step 0.5 nm, and time-per-point 0.5 s) with baseline subtraction and adaptive sampling enabled. Each sample was scanned 10 times and the traces were smoothed with a window scale of five and averaged. CD signals were converted from millidegree to molar ellipticity to account for small differences in enzyme concentrations (Figure S1). For the experiment where CD spectra were collected at different pH values, 50 mM potassium phosphate was used for pH 6.0–8.0 and 50 mM borate was used for pH 8.5–10.0 (Figure S2).

### 2.3 Preparation of CdRP

CdRP was prepared based on a previous method.^30^d-Ribose-5-phosphate disodium salt (1.0 mmol, Sigma-Aldrich) in H_2_O (1.0 mL) was added to 2.0 mL of ethanol followed by anthranilic acid (2.0 mmol) in ethanol (2.0 mL). The mixture was stirred at room temperature in the dark for 3 h, and the product oil was washed three times with ethyl acetate and triturated with ethanol. The solid product was collected by centrifugation and dried under a vacuum at room temperature to yield a pale yellow powder. The identity and purity of CdRP samples were verified using a Shimadzu HPLC-MS system equipped with a C18 column (Restek) and a mobile phase of 1:1 MeOH/10 mM ammonium formate in 0.15% formic acid. CdRP concentration was determined by measuring absorbance at 278 and 327 nm before and after irreversibly converting CdRP to IGP using *T*. *maritima* IGPS as previously described by Sterner and co-workers.^31^

### 2.4 Enzyme Activity Assays

Steady-state kinetics for *Mt*IGPS were determined by measuring the absorbance increase at 278 nm (ε = 5500 M^–1^ cm^–1^)^32^ in a Synergy H1 microplate reader at 25 °C in 100 mM PIPES, 2 mM DTT, pH 7.5. CdRP concentration in the well was 0–80 μM for wild-type and most mutants, and enzyme concentration was 0.05–60 μM (Figures S3–S12). Controls without enzyme and substrate were measured during each assay. Initial rate data were fit to the Michaelis–Menten equation by using nonlinear regression with KaleidaGraph. Note that in this paper we use the rate and initial velocity interchangeably. Due to its low activity, Lys119Arg assays utilized fluorescence measurements (ex. 278 nm/em. 340 nm) with a Synergy H1 microplate reader at 25 °C in 100 mM PIPES, 2 mM DTT, pH 7.5. A standard curve for the CdRP to IGP differential emission intensity was obtained using *T*. *maritima* IGPS to completely convert known concentrations of CdRP to IGP and record the change in fluorescence intensity. Wild-type *Mt*IGPS *k*_cat_ was measured via both absorbance and fluorescence to ensure the two protocols provided consistent results.

### 2.5 Rate versus pH Profiles

*Mt*IGPS and CdRP were mixed in MTEN buffer (50 mM MES, 25 mM Tris base, 25 mM ethanolamine, 100 mM NaCl, 2 mM DTT) at different pH values, and the rates of fluorescence intensity increase (ex. 278 nm/em. 340 nm) corresponding to IGP formation were recorded. The experiments were conducted under conditions where the initial velocities are expected to be governed by *k*_cat_ because the CdRP concentration (60 μM) was maintained at greater than *K*_M_ for wild-type and all mutants. The resulting rates were normalized by dividing all rates by the maximum rate measured in a given trial. Rate versus pH data displayed a bell-shaped curve and were fit with eq 1 where *v* = normalized rate, *C* = normalized pH-independent rate, and p*K*_a1_ and p*K*_a2_ are the acidity constant values associated with the ascending and descending portions of the curve, respectively.
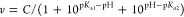
1

### 2.6 Solvent Deuterium Kinetic Isotope Effects and Solvent Viscosity Effects

Solvent deuterium kinetic isotope effects (SDKIEs) for *k*_cat_ were measured under a saturating concentration of CdRP (60 μM) at 25 °C in 100 mM PIPES, 2 mM DTT, pH 7.5, with 0.1 μM wild-type *Mt*IGPS, varying the percent of D_2_O. pD values were determined by measuring the pH using the equation pD = pH + 0.4. The SDKIE is defined as *k*_H_2_O_/*k*_D_2_O_.

The solvent viscosity effect (SVE) was determined by mixing *Mt*IGPS (0.1 μM) and CdRP (60 μM) in 100 mM PIPES, 2 mM DTT, pH 7.5, at different glycerol percentages (volume/volume). The fluorescence increase (ex. 278 nm/em. 340 nm) was measured at 25 °C. Control experiments were conducted using the same settings and conditions with the exception that the substrate or enzyme was eliminated from the reaction. The viscosities of the buffers with volume/volume percentages of glycerol were measured using an Ostwald viscometer.

### 2.7 Software Modeling

Because no CdRP-bound *Mt*IGPS structure is available in the Protein Data Bank, we used Autodock Vina^33^ and the structure of *Mt*IGPS bound with IGP and anthranilic acid (PDB 3T44) to model the binding of CdRP in *Mt*IGPS.

## 3 Results and Discussion

### 3.1 Steady-State Kinetics and Rate–pH Profile of Wild-Type *Mt*IGPS

To establish a baseline for our wild-type enzyme, we determined its steady-state parameters. We determined a *K*_M_ of 6.9 ± 1.4 μM (25 °C, pH 7.5) for *Mt*IGPS (Figure S3), which is lower than values reported by Czekster et al. (55 ± 3 μM, 25 °C, pH 7.5), Yang et al. (∼500 μM, 37 °C, pH 7.0), and Shen et al. (1.13 mM).^17,24,29^ Czekster et al. attributed the inconsistency among their value, and Yang’s and Shen’s *K*_M_ values to differences in the purity of CdRP, a hygroscopic and unstable substrate that easily decomposes and is prepared by each laboratory with variation in protocols.^34^ Impurities potentially present in substrate mixtures such as unreacted CdRP precursors (d-ribose-5-phosphate, anthranilic acid) and degradation products might compete with CdRP for binding and/or affect some measurements of CdRP concentrations. Additionally, Hankins et al. observed CdRP preparations containing material that quenches IGP fluorescence at concentrations over ∼50 μM and suggested that high purity CdRP is needed to study kinetics of IGPS enzymes with high *K*_M_ values.^35^ We used HPLC-MS to verify our substrate purity, and our wild-type kinetics utilized absorbance rather than fluorescence measurements. For comparison, other IGPS *K*_M_ values at 25 °C, pH 7.5, ranged from 0.006 μM for *T*. *maritima*, 0.085 μM for *S*. *solfataricus*, and 0.3 μM *for E*. *coli* to 255 μM for *S*. *cerevisiae*.^26^ Our *k*_cat_ for *Mt*IGPS (0.022 ± 0.002 s^–1^) is 7-fold lower than the *k*_cat_ of 0.16 ± 0.01 s^–1^ previously determined by Czekster and co-workers.^24^ Differences in the buffers and protein concentration determination methods used in the two studies could contribute to this discrepancy. Our *k*_cat_/*K*_M_ of 0.0032 ± 0.0007 s^–1^ μM^–1^ (25 °C, pH 7.5) for *Mt*IGPS is similar to *k*_cat_/*K*_M_ of 0.0029 s^–1^ μM^–1^ (25 °C, pH 7.5) determined by Czekster because both the *k*_cat_ and *K*_M_ determined by Czekster are higher than ours.

**Figure 2:**
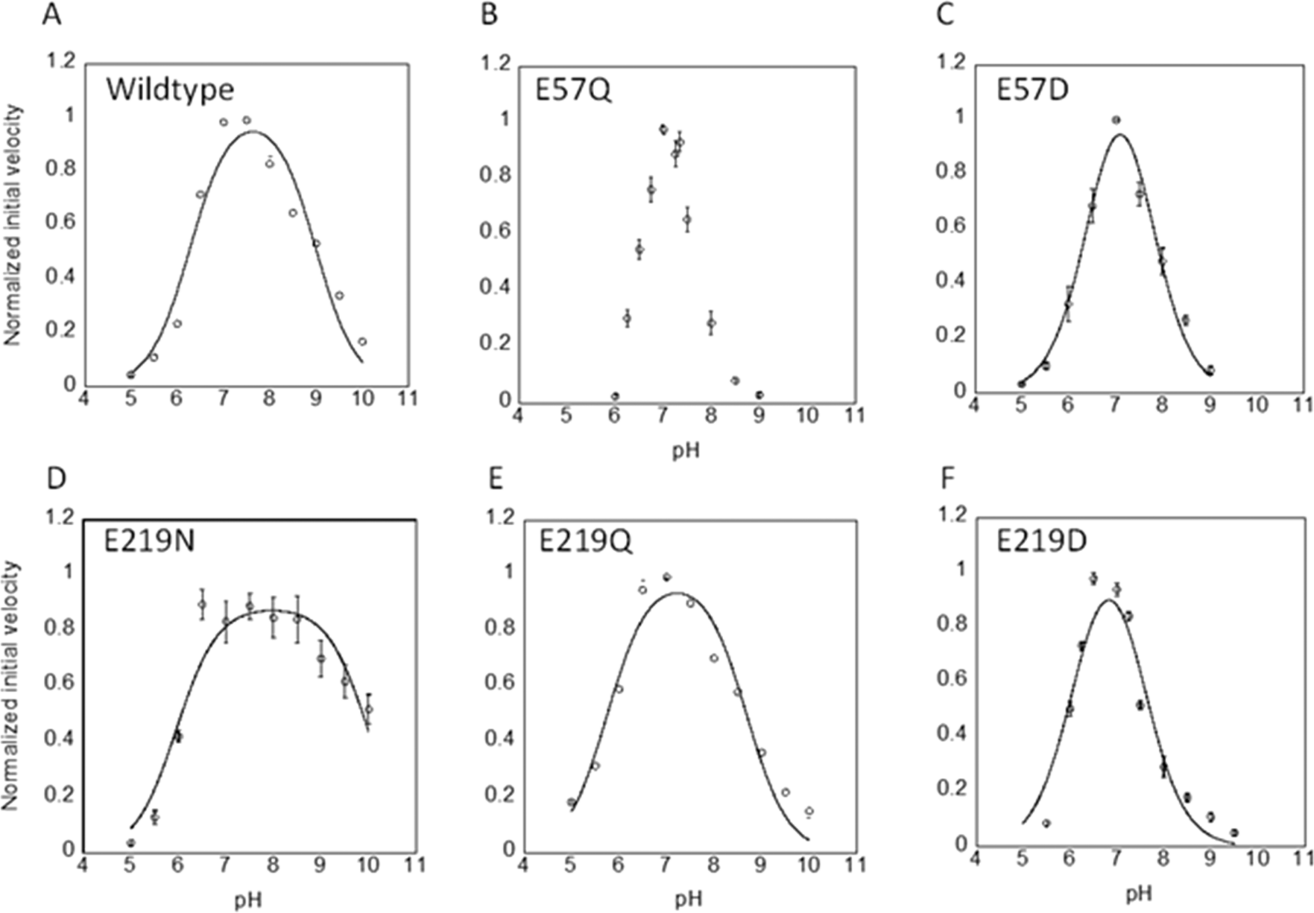
Mutations alter wild-type p*K*_a2_ values. Bell-shaped curves were obtained at 25 °C with 60 μM CdRP for (A) wild-type *Mt*IGPS at an enzyme concentration of 0.25 μM (*n* = 4), (B) Glu57Gln (20 μM; *n* = 7), (C) Glu57Asp (1 μM; *n* = 4), (D) Glu219Asn (1 μM; *n* = 7), (E) Glu219Gln (0.25 μM; *n* = 3), and (F) Glu219Asp (10 μM; *n* = 5). The experiments were conducted under conditions where the initial velocities are expected to be governed by *k*_cat_ because the CdRP concentration (60 μM) was maintained above *K*_M_ for wild-type and all mutants. The error bars represent standard errors. The experiments were performed using a minimum of two biological replicates per *n* total technical replicates; *n* is indicated above in parentheses for each experiment. Note that the rate versus pH data for Glu57Gln *Mt*IGPS did not yield a good fit with eq 1 (section 2.5) with a maximum normalized rate close to 1.0, and no fit is shown for this mutant.

Initial velocity versus pH plots give evidence for the existence of catalytic proton transfers and can help identify active site residues involved.^36^ Fitting the data as described in section 2.5 yields apparent p*K*_a_ values of the acids and/or bases involved as reported in Table 1. Yang et al. reported a bell-shaped curve for *Mt*IGPS with maximum activity at pH 7.0 (5 mM Tris-HCl buffers) and concluded that this indicates the presence of both a general acid and base (see Figure 1 for proposed mechanism).^29^ In contrast, Czekster et al. used a mixed buffer system (100 mM citrate and 100 mM HEPES) and reported a curve characterized by an ascending limb with p*K*_a_ = 6.0 ± 0.3 and a plateau at higher pHs, indicative of the involvement of only a catalytic base.^24^ Citrate and HEPES have relatively low p*K*_a_ values, and it is possible that the higher pH values were beyond the buffer system’s buffering range. We investigated the *Mt*IGPS rate versus pH profile using the MTEN buffer system, which covers the pH range and has minimal changes in ionic strength with pH. We observed a bell-shaped curve for *Mt*IGPS with maximum activity at pH = 7.5 with a p*K*_a1_ of 6.3 ± 0.1 and a p*K*_a2_ of 9.0 ± 0.1 (Figure 2A). Zaccardi et al. reported a similar curve for *Sulfolobus solfataricus* IGPS (*Ss*IGPS) with a p*K*_a1_ of 5.4 ± 0.2 and a p*K*_a2_ of 8.9 ± 0.2.^21^ As a control experiment, we recorded the CD spectra of *Mt*IGPS at pH values ranging from 6 to 10. The data indicate some change in secondary structure content at pH = 6.0 (Figure S2). Thus, we interpret the descending limb as evidence of the involvement of a general acid (Figure 1). The ascending limb may be reporting on the p*K*_a_ of a general base, but this aspect is unclear based on our CD data and deserves further investigation.

**Table 1:** Steady-State Kinetic Values and Acidity Constants (p*K*_a1_, p*K*_a2_) for Wild-Type and Mutant *Mt*IGPSs and Ratios of *k*_cat(mutant)_/*k*_cat(wild-type)_^a^

*Mt*IGPS	*k*_cat_ (s^–1^)	*K*_M_ (μM)	*k*_cat(mutant)_/*k*_cat(wild-type)_	p*K*_a1_	p*K*_a2_
wild-type	0.022 ± 0.002	6.9 ± 1.4	1.0	6.3 ± 0.1	9.0 ± 0.1
Glu57Ala	b	b	b	b	b
Glu57Asp	0.0078 ± 0.0009	12 ± 4	0.35	6.5 ± 0.1	7.7 ± 0.1
Glu57Gln	1.10 × 10^–4^ ± 0.05 × 10^–4^	4.5 ± 0.8	0.0050		
Lys59Ala	b	b	b	b	b
Lys59Arg	4.8 × 10^–5^ ± 0.2 ×10^–5^	19 ± 2	0.0022	6.4 ± 0.1	8.5 ± 0.1
Lys119Ala	b	b	b	b	b
Lys119Arg	8.1 × 10^–6^ ± 0.1 × 10^–6^	5.1 ± 0.7	0.00037	7.3 ± 0.1	8.7 ± 0.1
Glu168Ala	b	b	b	b	b
Glu168Asp	0.0110 ± 0.0003	26 ± 3	0.50	6.8 ± 0.2	9.0 ± 0.2
Glu168Gln	2.3 × 10^–6^ ± 0.1 × 10^–6^	2.4 ± 0.3	0.00010	7.0 ± 0.1	8.3 ± 0.1
Glu219Ala	b	b	b	b	b
Glu219Asp	2.70 × 10^–4^ ± 0.02 × 10^–4^	3.9 ± 0.3	0.012	6.1 ± 0.1	7.6 ± 0.2
Glu219Asn	0.0103 ± 0.0007	18 ± 3	0.47	6.0 ± 0.1	10.0 ± 0.2
Glu219Gln	0.23 ± 0.02	21 ± 4	10.5	6.2 ± 0.1	9.9 ± 0.1

a Data are presented with standard errors (SE). Lys119Arg and Glu168Gln values are based on fluorescence measurements. All rate versus pH profiles showed bell-shaped curves, and p*K*_a1_ and p*K*_a2_ are shown with SEs. Note that the rate versus pH data for Glu57Gln *Mt*IGPS did not yield a good fit with eq 1 (section 2.5) with a maximum normalized rate close to 1.0 and p*K*_a_ values are not included for this mutant.

b We observed no activity for the alanine variants.

### 3.2 Steady-State Kinetic Parameters of *Mt*IGPS Mutants and *Mt*IGPS Ligand Interactions

Since Lys59 and Lys119 in *Mt*IGPS may serve as catalytic acids (see Introduction, section 1), we initially prepared the alanine mutants of both. We did not detect any activity in either our Lys59Ala or our Lys119Ala variants (Table 1), emphasizing the key catalytic roles of these residues and adding new information to the existing data obtained with other homologues.

We also mutated the three glutamic acid residues discussed in the Introduction, section 1, (Glu57, Glu168, and Glu219) to alanine and observed no activity for any of the mutant enzymes (Table 1), consistent with each of these residues also playing a very important functional role. The Glu168Ala mutation in *Mt*IGPS was previously reported by Shen et al. as having an increased *K*_M_ over wild-type.^17^ They did not report *k*_cat_ or *V*_max_ values, but the *K*_M_ value indicates that they observed some activity for this mutant while we did not.

Given the dramatic impact of our alanine mutations on catalysis, we proceeded with more conservative mutations, replacing the three glutamic acids (Glu57, Glu168, and Glu219) with both aspartic acid and glutamine, and we also made Glu219Asn. We mutated both Lys59 and Lys119 to arginine. Mutations at four of the sites we studied (Glu57, Lys59, Lys119, and Glu219) have never before been reported in *Mt*IGPS, and only the alanine mutant has been previously reported for Glu168.^17^ Steady-state kinetic parameters obtained for all of these mutants are shown in Table 1. CD spectra demonstrate that all mutants except Glu219Gln have secondary structure contents similar to wild-type at pH 7.5, suggesting that the variations in kinetic parameters at pH 7.5 are not due to significant protein folding differences (Figure S2). Additional Glu219Gln structural analysis in the future should reveal the explanation for its unique CD spectrum.

We found a >2700-fold reduction in *k*_cat_ with our Lys119Arg mutant compared to wild-type (Figure S7). These data continue to indicate Lys119’s critical role, likely as the general acid, as proposed by Zaccardi et al. and Hennig et al. in *Ss*IGPS and Darimont et al. in *E*. *coli* IGPS.^21−23^ Studies involving the corresponding residues were conducted in *E*. *coli* IGPS showing that Lys114 (corresponding to Lys119 in *Mt*IGPS) is an essential amino acid in *E*. *coli* IGPS.^22^ The Lys59Arg variant has a 460-fold reduction in activity compared to the wild-type enzyme, indicating that Lys59 also plays an important role in *Mt*IGPS catalysis. *Mt*IGPS maintains some activity when these lysine amino groups are replaced with guanidine but is greatly compromised.

Our data show that Glu219Gln *Mt*IGPS has 10-fold enhanced catalytic activity relative to wild-type (Table 1, Figures S3 and S9), similar to Zaccardi’s observation with Glu210Gln in *Ss*IGPS.^21^ Glu and Gln side chains both can form hydrogen bonds, but only Glu can be charged (anion) or act as a base. Therefore, our data rule Glu219 out as the active site base in *Mt*IGPS given no reduction in activity with the Glu to Gln replacement. This finding is in contrast to the proposal by Mazumder-Shivakumar et al.^20^ for the role of Glu210 in *Ss*IGPS. Interestingly, our data show an 81-fold reduction in catalytic activity for Glu219Asp and a 2-fold reduction for Glu219Asn (Table 1, Figures S10 and S11). To the best of our knowledge, the corresponding Glu to Asp and Glu to Asn mutations have not been previously reported in any IGPS enzyme.

**Figure 3:**
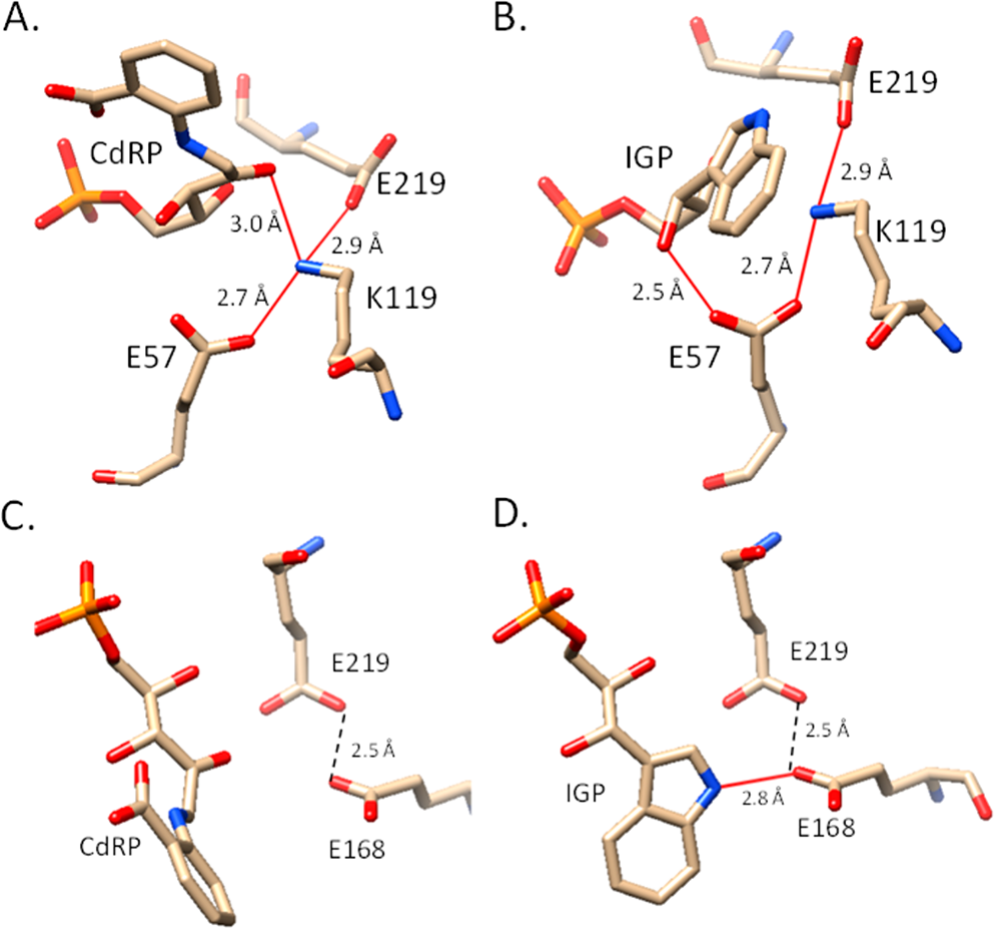
Structural orientation of *Mt*IGPS Glu219, Lys119, and Glu57 with (A) CdRP and (B) IGP, and (C) Glu168 and Glu219 with CdRP and (D) IGP. Graphics correspond to the original (IGP-bound) and Autodock Vina docked structure of CdRP in the active site of PDB 3T44*Mt*IGPS. Red lines represent hydrogen bonds predicted by Chimera, and black dashes represent distances. The figures were prepared with Chimera.^38^

To understand interactions of Glu219 with other active site residues, we wanted to examine both CdRP and IGP bound complexes of *Mt*IGPS. However, no structures of *Mt*IGPS bound to CdRP (or rCdRP, the reduced substrate analog which is not turned over)^37^ are in the Protein DataBank. We used Autodock Vina^33^ and the available structure of *Mt*IGPS (PDB 3T44) bound with both the enzymatic product IGP and anthranilic acid to model the binding of CdRP to *Mt*IGPS. The four most stable results (or poses) produced by the software upon docking CdRP into the active site had very similar energy scores. Of these four lowest-energy poses, we focused on the one that had the best alignment between the aromatic ring portion of the docked CdRP and the corresponding anthranilic acid present in the crystal structure (PDB 3T44). This model was used to prepare Figure 3A to show the orientation of *Mt*IGPS Glu219, Lys119, Glu57 with CdRP. Figure 3A shows the Glu219 carboxylate positioned to interact with the side chain of Lys119 (O to N distance 2.9 Å) via either a hydrogen bond or a salt bridge. Figure 3B shows that this interaction between Glu219 and Lys119 side chains is maintained at the same O to N distance of 2.9 Å in the complex with IGP bound.

The simulations of Mazumder-Shivakumar and Bruice led them to propose that Glu210 orients and/or reprotonates Lys110 in *Ss*IGPS,^20^ and this is consistent with our data in *Mt*IGPS showing that side chain length and the ability to donate/accept protons matters in this position. Thus, we hypothesize that Glu219 interacts with and orients Lys119 in *Mt*IGPS and that a shorter Asp side chain negatively affects this interaction. This hypothesis is also consistent with the enzyme tolerating the Glu219Gln mutation as these side chains have a similar length and Gln can also participate in hydrogen bonding. The wild-type and Glu219Asn, Glu219Asp, and Glu219Gln variants together provide us with a better two-point characterization of position 219. First, an amide side chain (Gln or Asn) at this position results in higher catalytic activity relative to the carboxylic acid (Glu or Asp) with the same side chain length; the uncharged amide is preferred to the carboxylic acid. Second, a longer side chain (Glu or Gln) results in higher catalytic activity compared to the shorter side chain with the same functional group (Asn or Asp), apparently due to more optimal positioning.

The Glu57Gln mutation resulted in a 200-fold reduction in *k*_cat_, while the *k*_cat_ of Glu57Asp is only 3-fold reduced, indicating that the Asp side chain is better tolerated as a replacement for Glu at position 57 compared to Gln (Table 1, Figures S4 and S5). The smaller differences between wild-type and Glu57Asp *Mt*IGPS *k*_cat_ values underscore the importance of maintaining a charged carboxylate in this position, which we believe is important for stabilizing Lys119 in its protonated ammonium form.

The activity of Glu168Gln *Mt*IGPS is >9000-fold lower than wild-type (Table 1). The equivalent mutation Glu163Gln in *E*. *coli* IGPS resulted in a 540-fold reduction in activity.^22^ The Glu168Asp *Mt*IGPS *k*_cat_ is reduced only 2-fold versus wild-type (Table 1, Figure S8). Glu168Asp *Mt*IGPS also has an elevated *K*_M_ (26 ± 3 μM compared to 6.9 ± 1.4 μM for wild-type) indicating a lower CdRP affinity for the mutant (Figure S8). The anionic Glu168 is 2.8 Å from the nitrogen atom in IGP (Figure 3D). Glu168 is a potential catalytic base in *Mt*IGPS, and based on our data, we hypothesize that an additional (or even primary) role of Glu168 is to stabilize the ammonium cation expected to develop in **I**_**1**_ and thus facilitating catalysis by lowering the barrier to formation of this intermediate in which aromatic resonance energy is lost (Figure 1). This explains why Glu168Asp retains significant activity as it presents a similar but less optimally positioned charge relative to the substrate. Studies involving the corresponding residues in *E*. *coli* IGPS show that Glu163 (corresponding to Glu168 in *Mt*IGPS, Figure S13) is an essential amino acid in *E*. *coli* IGPS.^22^

Zaccardi and co-workers proposed a mechanism for *Ss*IGPS in which Lys110 (Lys119 in *Mt*IGPS) serves as the catalytic acid in the formation of **I**_**1**_ and then a different residue (Lys53) is the proton source for the subsequent dehydration step (Figure 1).^21^ In such a mechanism, both acids would have to be reprotonated prior to the next catalytic cycle. This could be done by water within the active site after IGP release or a nearby residue. Other proposed IGPS mechanisms implicate a single-acid model in which Lys119 (*Mt*IGPS numbering) would have to be reprotonated twice during the catalytic cycle. Mazumder-Shivakumar and Bruice suggested based on molecular dynamics simulations that *Ss*IGPS Glu210 (Glu219 in *Mt*IGPS) serves as the catalytic base during the dehydration step and then transfers its proton to the single catalytic lysine acid allowing it to function again for dehydration.^20^ Our data do not support such a role for Glu219 in *Mt*IGPS because our Glu219Gln mutant has a 10-fold higher activity versus the wild-type, and the Gln side chain amide cannot protonate Lys119. However, Glu57 in *Mt*IGPS is in position to function in this role and reprotonate Lys119 (Figure 3A).

### 3.3 Initial Velocity–pH Profiles of *Mt*IGPS and Mutant Enzymes

Our rate versus pH profiles for wild-type and mutant *Mt*IGPS enzymes are bell-shaped. This shape is consistent with the operation of both a catalytic base (ascending limb) and an acid (descending limb) (Figure 1). There are relatively small variations among the wild-type and mutant p*K*_a1_ values obtained by fitting the ascending curves (Figures 2 and S14–S17, and Table 1). However, the differences we observe in CD data for wild-type *Mt*IGPS at pH 6.0 versus pH 7.0 and higher (Figure S2) give rise to the possibility of pH-dependent structural effects, and it is in doubt whether the calculated p*K*_a1_ values are actually correlated to the active site catalytic base. Accordingly, we focus our analysis on p*K*_a2_ values.

The Glu57Asp and Glu219Asp mutants have p*K*_a2_ values determined from the descending limbs of their profiles of nearly 1.5 p*K*_a_ units lower than wild-type (Table 1, Figure 2). Our hypothesis of Lys119 as the catalytic acid in the cyclization step forming **I**_**1**_ in *Mt*IGPS is consistent with our data in Table 1 and findings in other IGPS homologues, in which case the p*K*_a2_ values correlate to the Lys119 side chain ammonium ion. The Glu57 and Glu219 side chains interact with the side chain of Lys119 (Figure 3A,B). The negatively charged carboxylates can form stabilizing salt bridges with the ammonium form of Lys119. In Glu57Asp, the shorter Asp side chain is not expected to be as well positioned as the longer Glu to form the optimal salt bridge with Lys119, and p*K*_a2_ is lowered accordingly. The Glu219 side chain carboxylate is also in position to interact with Lys119 (Figures 3A and 3B) and affect this ammonium acid in a similar way as seen with the reduced p*K*_a2_ of Glu219Asp. The p*K*_a2_ values in Glu219Gln and Glu219Asn are both raised from wild-type by approximately 1.0 p*K*_a_ unit, and we plan to investigate this observation further in the future (Table 1 and Figure 2D,E). The data in Table 1 along with structural information in Figure 3 are consistent with a model in which the Glu57 and Glu219 side chains both impact the positioning and p*K*_a_ of the critical acidic Lys119 side chain.

### 3.4 Solvent Deuterium Kinetic Isotope and Solvent Viscosity Effects (SDKIE/SVE) in Wild-Type *Mt*IGPS

**Figure 4:**
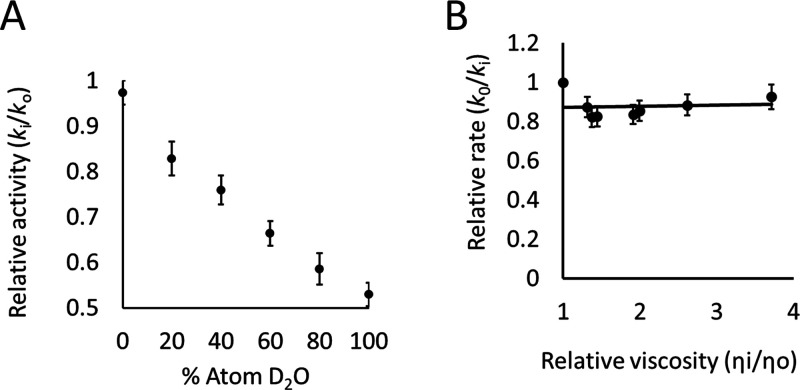
(A) *Mt*IGPS SDKIE (1.9 ± 0.1) was determined at 25 °C with 60 μM CdRP and an enzyme concentration of 0.1 μM (*n* = 11), conditions where the initial velocities are expected to be governed by *k*_cat_ because the CdRP concentration (60 μM) was maintained above the *K*_M_. The SDKIE is defined as *k*_H_2_O_/*k*_D_2_O_. (B) SVE on *k*_cat_ was determined using the same experimental conditions but with different glycerol percentages (v/v) (*n* = 12). The error bars represent standard errors. The experiments were performed using a minimum of two biological replicates per *n* total technical replicates; *n* is indicated above in parentheses for each experiment.

We observed an SDKIE on the steady-state turnover rate constant for *Mt*IGPS (Figure 4A). Consistent with work by Czekster et al., the steady-state turnover rate constant decreased with an increasing D_2_O fraction. Our SDKIE value for wild-type is 1.9 ± 0.1, similar to that determined by Czekster et al. (1.6 ± 0.1).^24^ These findings suggest that a solvent-exchangeable proton is transferred during the enzymatic rate-determining step and that the rate-determining step occurs after formation of the substrate–enzyme complex. As discussed by Zaccardi et al., the initial ring closure step (CdRP → **I**_**1**_ in Figure 1) is expected to be isotope-sensitive.^21^ From a mechanistic perspective, this step would be predicted to be the slowest and rate-determining one since it requires loss of aromaticity in forming **I**_**1**_ while the subsequent steps all involve re-forming a 6π aromatic and then the larger 10π ring system in IGP. This agrees with our measurements of the *Mt*IGPS steady-state turnover rate constant *k*_cat_ at different concentrations of glycerol. We did not observe a decreased rate under more viscous conditions, indicating that the viscosity-sensitive, diffusion-limited steps of substrate binding and product release are not rate-determining (Figure 4B).

## 4 Conclusions

We carried out mutagenesis studies in *Mt*IGPS involving five residues (Glu57, Lys59, Lys119, Glu168, and Glu219), which were predicted to be important for *Mt*IGPS function based on the broader IGPS literature and our own detailed review of IGPS structural information. Mutation of any of these amino acids to alanine results in the loss of all detectable activity, indicating the critical nature of each of these residues for *Mt*IGPS catalysis. The drastic losses in activity observed by multiple single mutations underscore the highly coordinated nature of the IGPS active site. Additionally, protein loop motions play an important role in IGPS catalysis during which part of the substrate must move from one binding pocket to another^18^ and studying the details and regulation of such dynamics in *Mt*IGPS remains a need and will be the subject of future work.

**Figure 5:**
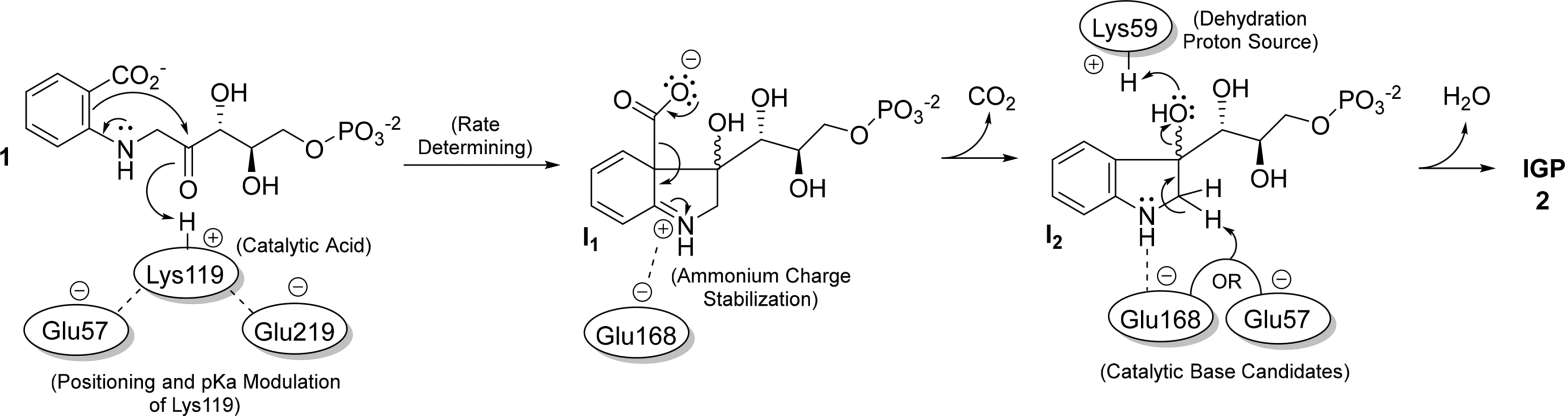
Model summarizing the roles of active site residues studied in the mechanism of *Mt*IGPS catalysis.

Our characterization of numerous more conservative mutations in addition to alanine combined with predictions of enzyme–substrate interactions provided by computer modeling yield new insights into these residues’ roles in *Mt*IGPS. We considered our results in context of Zaccardi’s mechanism for *ss*IGPS^21^ and summarized them in our new model for *Mt*IGPS catalysis shown in Figure 5. We propose that Lys119 is the catalytic acid in the cyclization step (CdRP → **I**_**1**_). Furthermore, Lys119, Glu57, and Glu219 appear to form a triad where Glu57 and Glu219 both modulate the p*K*_a_ of Lys119 and position it in the proper place for optimal catalysis (Figure 3A,B). The Glu57 side chain oxygen is located 2.7 Å from the Lys119 nitrogen, an appropriate distance for a strong salt bridge. The Glu219 side chain oxygen is only 2.9 Å from the Lys119 side chain nitrogen. Consideration of the chemistry involved along with our SDKIE and SVE results strongly indicates that this cyclization step forming **I**_**1**_ is rate-determining. Interestingly, this means that protonation of the CdRP carbonyl occurs in a concerted fashion along with the ring closure. Such a lack of activation (by protonation) of the carbonyl prior to attack lends support for our proposed novel role for Glu168 in facilitating this step by stabilizing the developing ammonium ion on the substrate during its conversion to **I**_**1**_ through a charge–charge interaction.

Decarboxylation and loss of CO_2_ from **I**_**1**_ re-establish a 6π aromatic system in **I**_**2**_ and set the stage for the dehydration step forming IGP. The dehydration is expected to be energetically favorable and driven by the formation of an expanded 10π aromatic indole ring system. We propose that this step occurs through either an E1- or E2-like mechanism depending on the relative timing of the deprotonation and breaking of the **I**_**2**_ C–O bond. Our model in Figure 5 shows an E2-like mechanism, but the benzylic nature of the carbon atom bonded to the oxygen being lost makes an E1-like mechanism possible as well. In either case, dehydration requires both an acid and a base. Based on our results from mutating Lys59 and Zaccardi’s work with *ss*IGPS,^21^ we propose that the proton source in the *Mt*IGPS dehydration step is Lys59. Our data rule out Glu219 as the general base but do not give us definitive evidence whether it is Glu57 or Glu168 serving this role in *Mt*IGPS. The side chain oxygen of Glu168 is indeed in optimal position to accept a proton from the adjacent ring carbon for dehydration (Figure 3C,D). Thus, Glu168 may function in stabilizing formation of the cation in **I**_**1**_ and then act as a dehydration base before cycling its proton prior to the next round of catalysis. The only 2-fold reduced catalytic activity of Glu168Asp, which retains the carboxylate functional group, and very low activity of Glu168Gln, which has no negative charge or basicity, are consistent with these roles. The location and activities of our Glu57Asp and Glu57Gln *Mt*IGPS mutants are consistent with this residue serving as the catalytic base as well. Additional structural and other data in the future will provide an even more detailed understanding of the dehydration step in *Mt*IGPS.

Glu219, while not the catalytic base, is still an important residue, and both its side chain length and charge greatly impact efficient *Mt*IGPS catalysis. The enhanced activity of our Glu219Gln *Mt*IGPS mutant is particularly intriguing. We believe that the interaction of one of the Glu219 side chain oxygen atoms with Lys119 affects the orientation and p*K*_a_ of this ammonium acid. Interestingly, the other Glu219 side chain oxygen is located 2.5 Å from one of the Glu168 side chain oxygen atoms, and thus additional interactions with Glu168 could affect catalysis. For example, the lack of a negative charge in Glu219Gln may relieve repulsion between the normally identically charged Glu219 and Glu168 side chains and therefore stabilize a deprotonated anionic Glu168, something necessary for its role in stabilizing **I**_**1**_ and as a potential catalytic base. There are other possibilities, including the possibility that the Glu219Gln mutation alters the conformational ensemble or flexibility of the enzyme. Additional experiments and efforts related to structure determination are underway to assess whether the mutation results in changes in the average conformation to understand the increased activity of Glu219Gln. Bacterial IGPS enzymes are highly conserved with regard to their active site sequences and structural homology.^26^ Our new findings implicate a complex and highly coordinated *Mt*IGPS active site structure with multiple critical residues and side chain interactions, all required for normal catalytic function.

## Supporting Information Available

CD spectra, steady-state kinetics, amino acid alignment for selected IGPS enzymes, and rate versus pH profiles (PDF)
