# Supplementary material for: Investigating the Roles of Active Site Residues in Mycobacterium tuberculosis Indole-3-glycerol Phosphate Synthase, a Potential Target for Antitubercular Agents
Source: ACS Bio Med Chem Au. 2023 Jul 26;3(5):438–47. doi: 10.1021/acsbiomedchemau.3c00029 (PMC10591298; doi:10.1021/acsbiomedchemau.3c00029)
Supplement: Supplementary file 1 — bg3c00029_si_001.pdf [file bg3c00029_si_001.pdf]

SUPPORTING INFORMATION

Investigating the roles of active site residues in *M. tuberculosis* indole-3-glycerol phosphate synthase, a potential target for anti-tubercular agents

David W. Konas, Sarah Cho, Oshane D. Thomas, Maryum M. Bhatti, Katherine Leon Hernandez, Cinthya Moran, Hedda Booter, Thomas Candela, Joseph Lacap, Paige McFadden, Savannah van den Berg, Alyssa M. Welter, Ashley Peralta, Cheryl A. Janson, Jaclyn Catalano, and Nina M. Goodey*

Department of Chemistry and Biochemistry, Montclair State University

1 Normal Avenue, Montclair, NJ, 07043, USA

*Corresponding author: Nina M. Goodey: Phone: (973) 666 1368;

E-mail: goodeyn@mail.montclair.edu

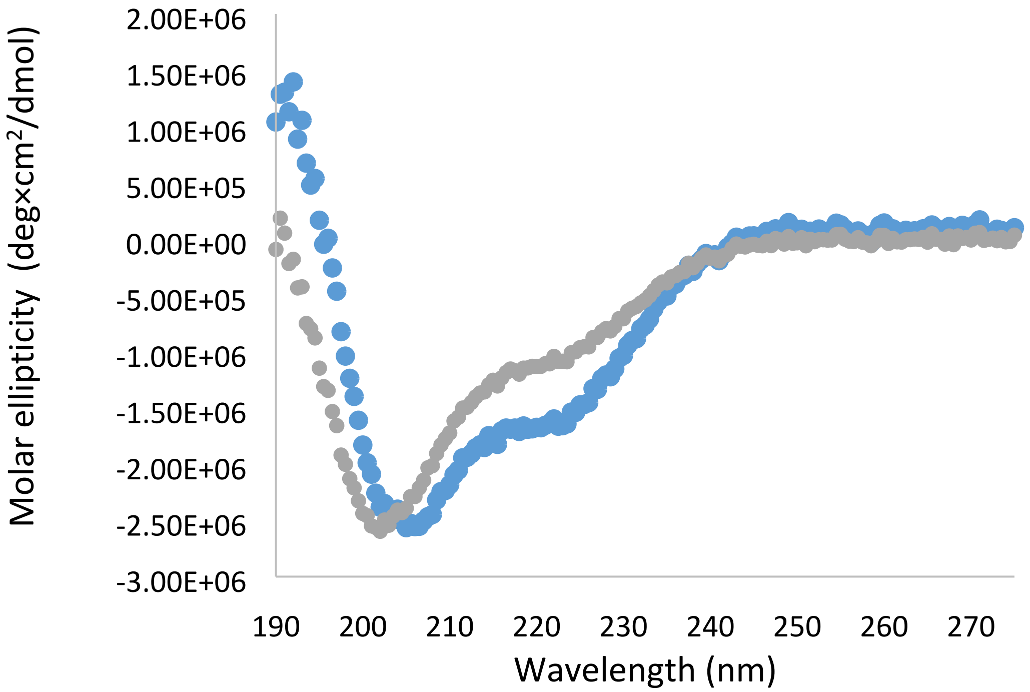

Fig. S1. Circular dichroism spectrum showing the difference in secondary structure between wildtype (gray) and Glu219Gln *Mt*IGPS (blue).

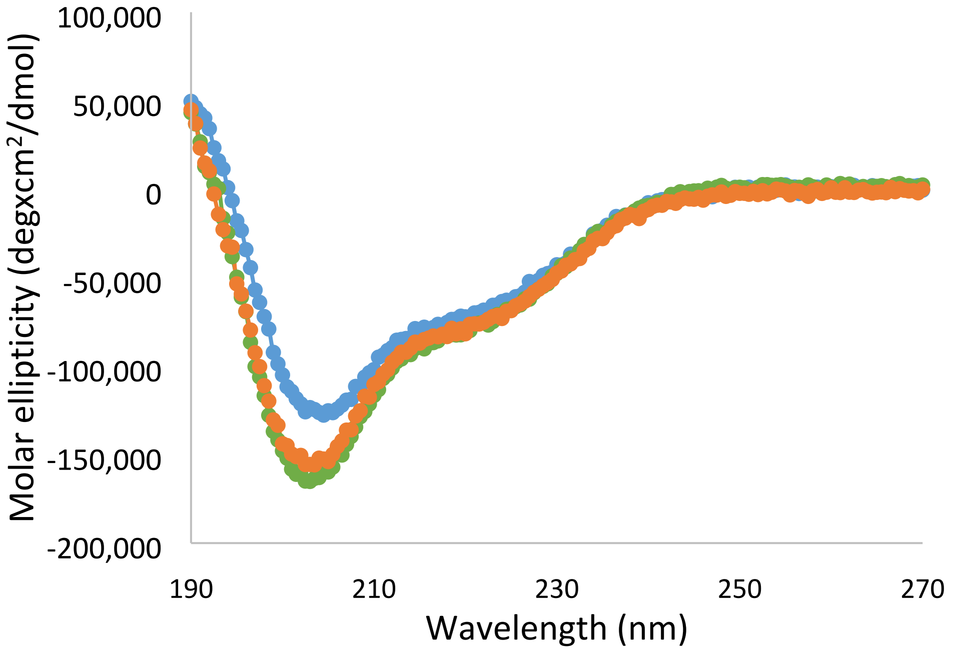

Fig. S2. Circular dichroism spectrum showing the difference in secondary structure between wildtype for pH 6 (blue), pH 7 (orange) and pH 8 (green).

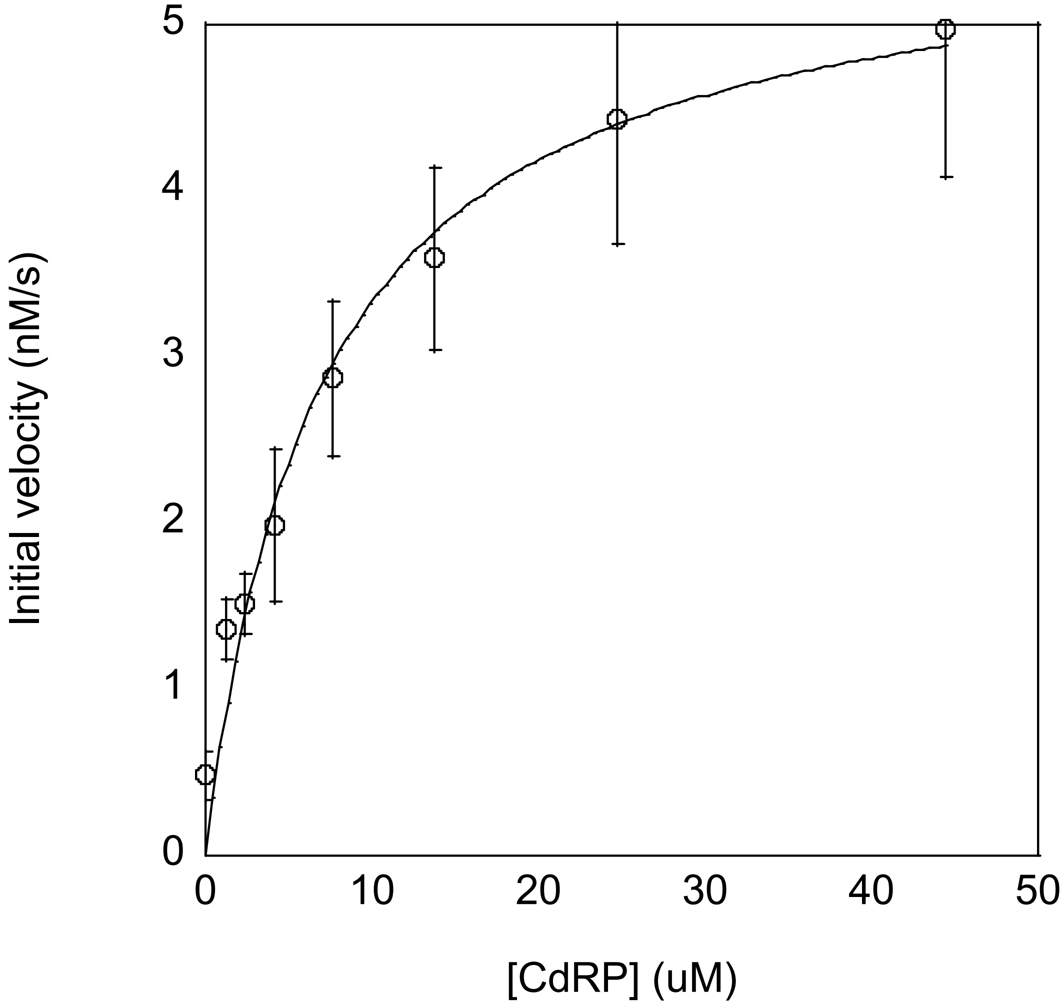

Fig. S3. Plot of wildtype *Mt*IGPS velocity in nM/s graphed against CdRP concentration. Each data point represents an average of five measurements at pH 7.5 and 0.25 μM enzyme. The data were fitted to the Michaelis-Menten equation to obtain a k_{cat} of 0.022 ± 0.002 s^{-1} and a K_M of 6.9 ± 1.4 μM (SE).

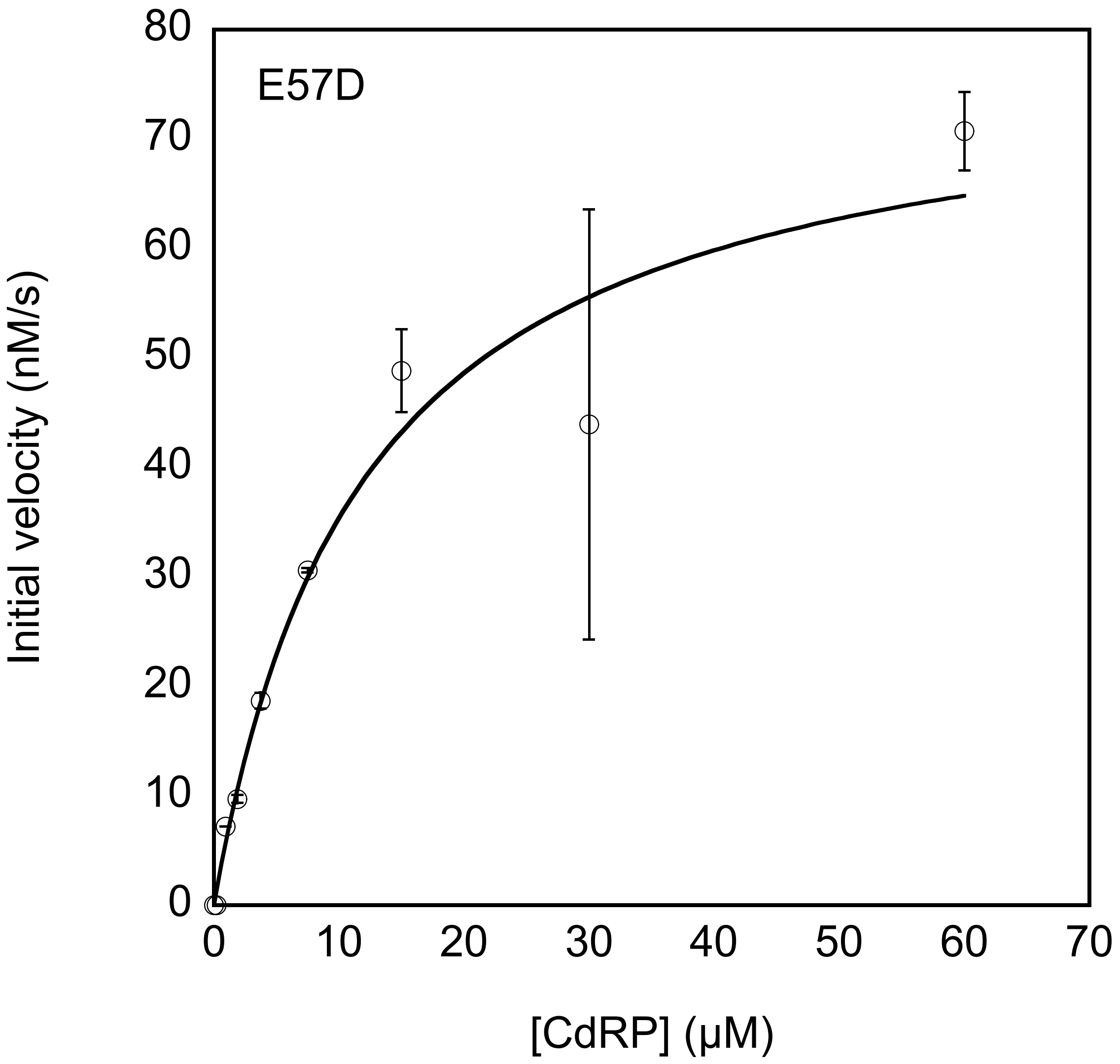

Fig. S4. Plot of Glu57Asp *Mt*IGPS velocity in nM/s graphed against CdRP concentration (μM) obtained at pH of 7.5 and 10 μM enzyme concentration. Each data point represents an average of two measurements with SEs and the data were fitted to the Michaelis-Menten equation with k_{cat} of 0.0078 ± 0.0009 s^{-1} and K_M of 12 ± 4 μM.

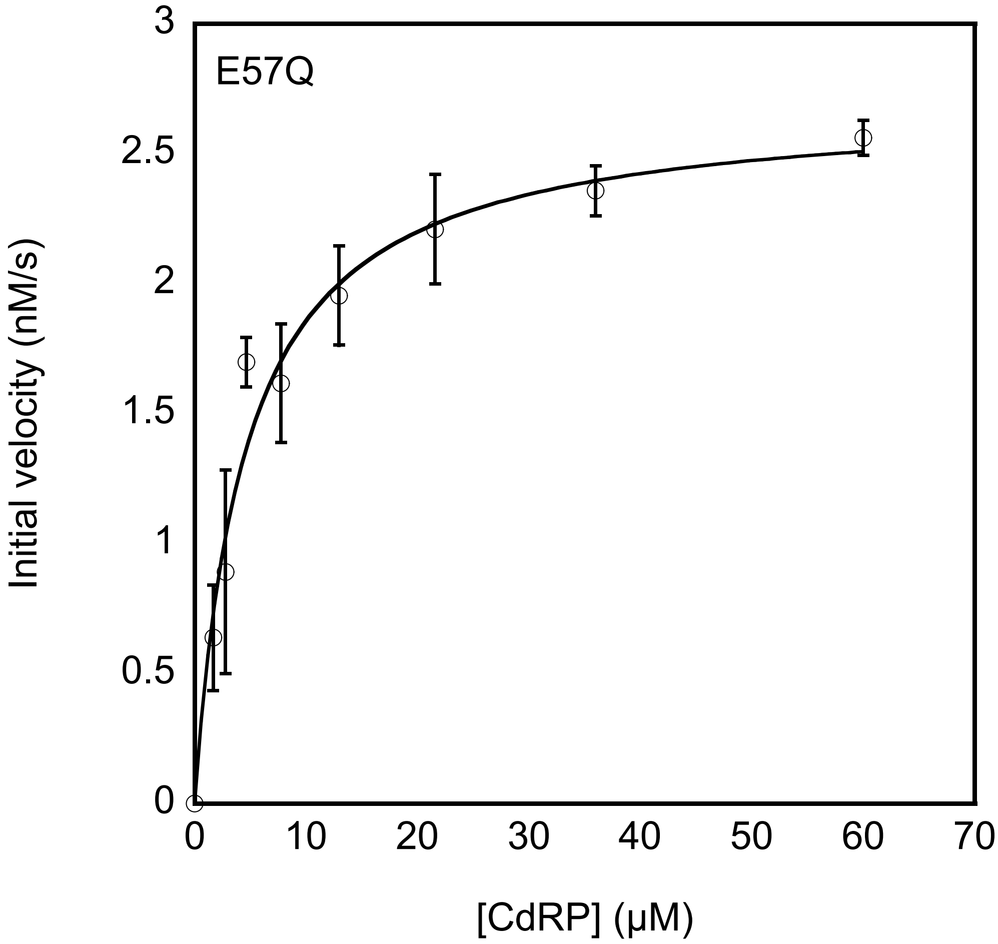

Fig. S5. Plot of Glu57Gln *Mt*IGPS velocity in nM/s graphed against CdRP concentration (µM) obtained at pH of 7.5 and 24.8 µM enzyme concentration. Each data point represents an average of three measurements with SEs and the data were fitted to the Michaelis-Menten equation with k_{cat} of $1.10 \times 10^{-4} \pm 0.05 \times 10^{-4}$ s^{-1} and K_M of 4.5 ± 0.8 µM.

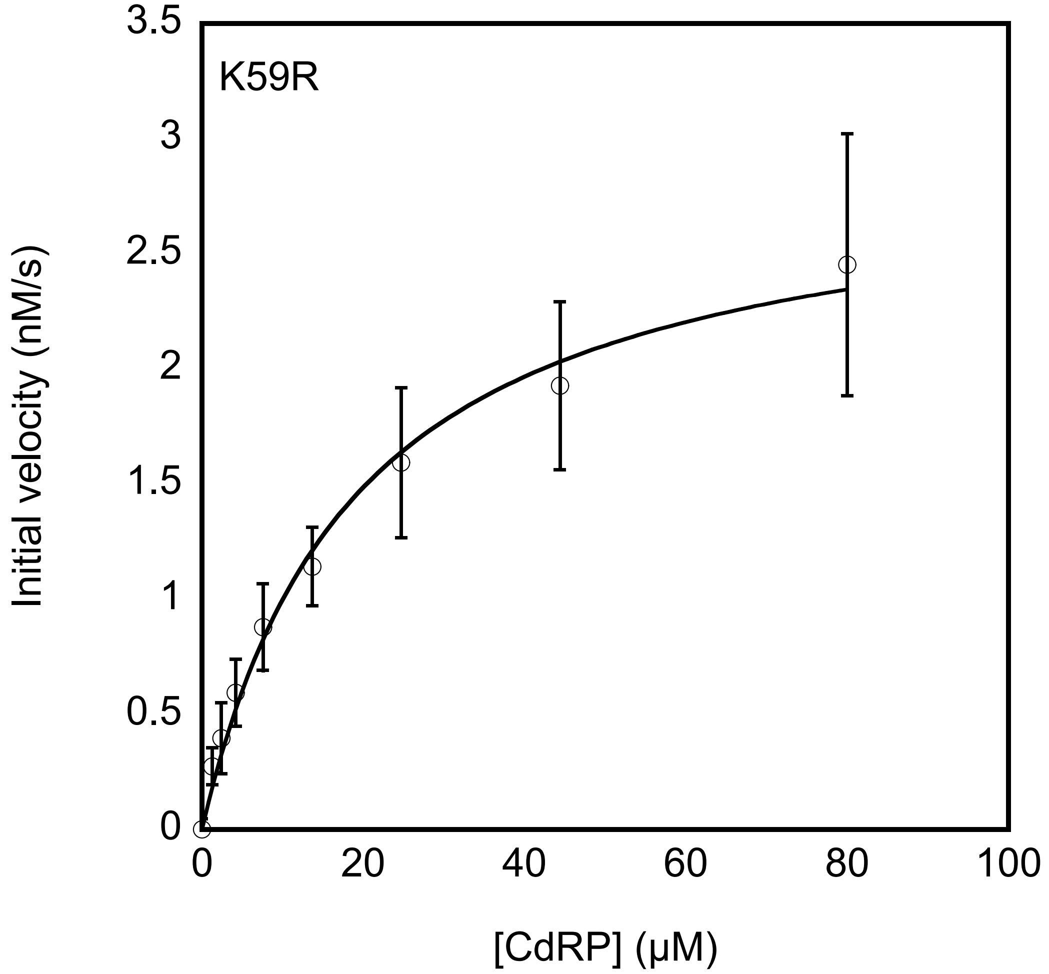

Fig. S6. Plot of Lys59Arg *Mt*IGPS velocities in nM/s graphed against CdRP concentration in µM obtained at pH of 7.5 and 60 µM enzyme concentration. Each data point represents an average of three measurements with SEs and the data were fitted to the Michaelis-Menten equation to obtain a k_{cat} of $4.8 \times 10^{-5} \pm 0.2 \times 10^{-5}$ s^{-1} and a K_M of 19 ± 2 µM.

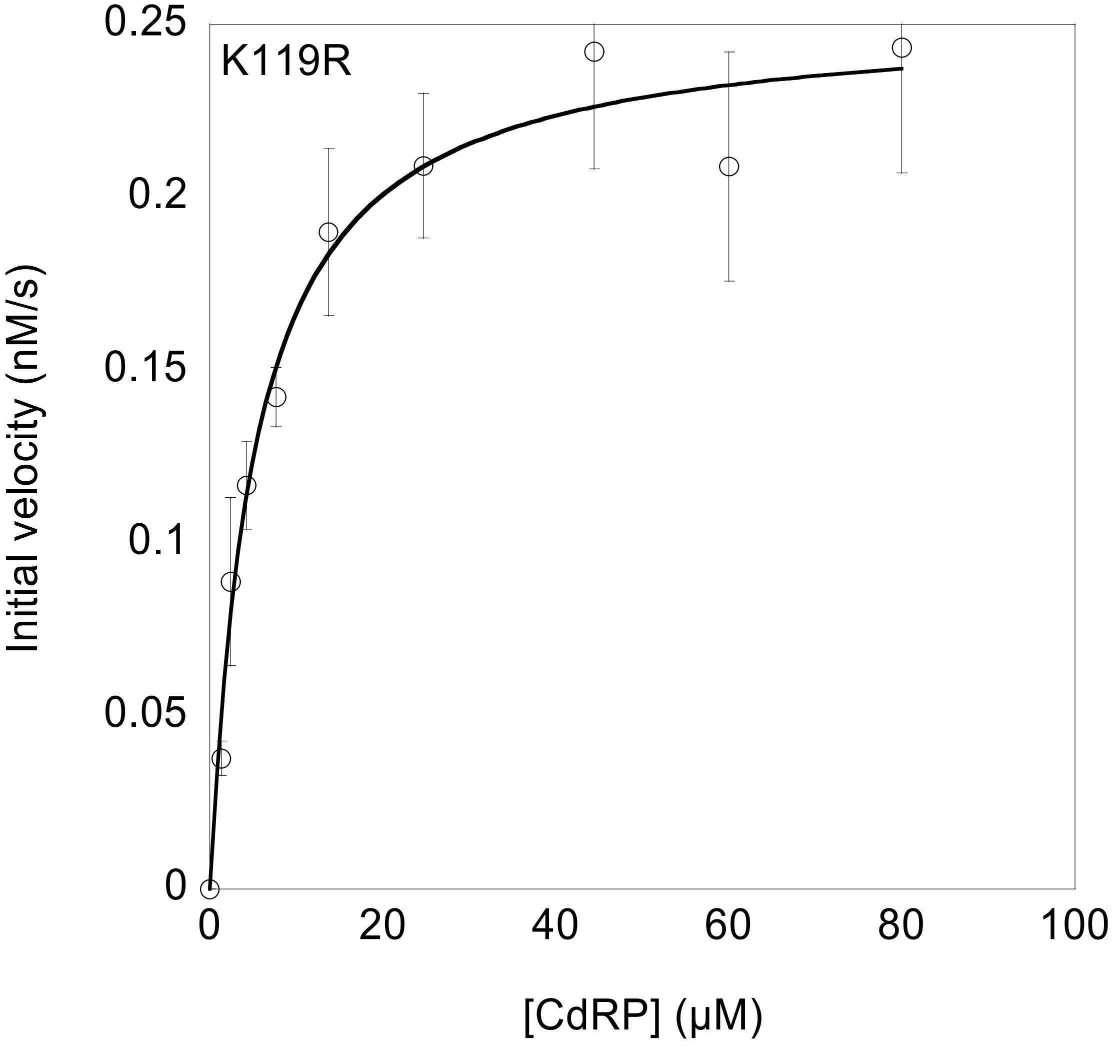

Fig. S7. Plot of Lys119Arg *Mt*IGPS velocities in nM/s graphed against CdRP concentration in μM obtained at pH of 7.5 and 60 μM enzyme concentration. Each data point represents an average of three measurements with SEs and the data were fitted to the Michaelis-Menten equation to obtain a k_{cat} of $8.1 \times 10^{-6} \pm 0.1 \times 10^{-6}$ s^{-1} and a K_M of 5.1 ± 0.7 μM.

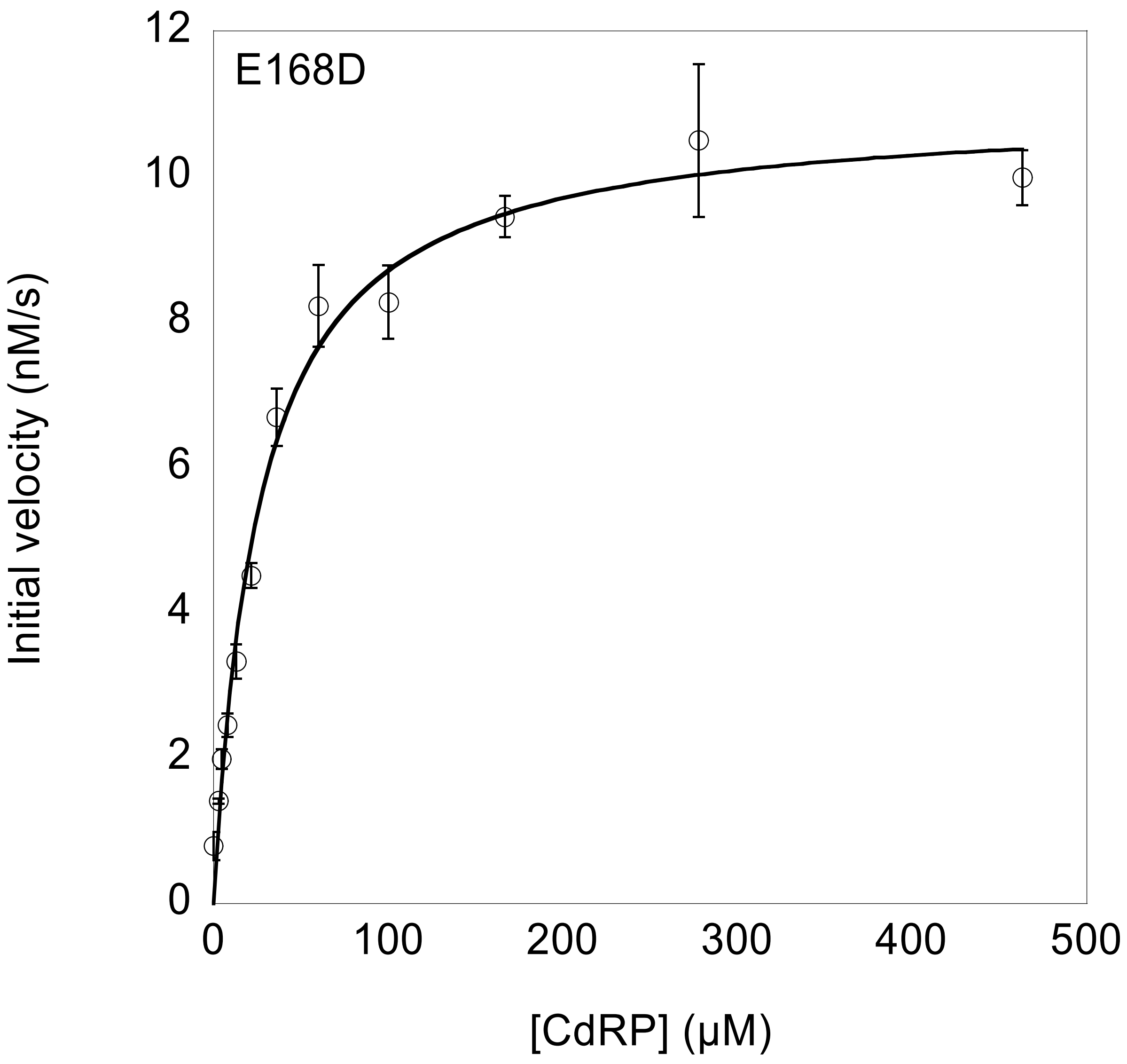

.

Fig. S8. Plot of Glu168Asp *Mt*IGPS velocity in nM/s graphed against CdRP concentration (μM) obtained at pH of 7.5 and 1 μM enzyme concentration. Each data point represents an average of three measurements with SEs and the data were fitted to the Michaelis-Menten equation with k_{cat} of 0.0110 ± 0.0003 s^{-1} and K_M of 26 ± 3 μM.

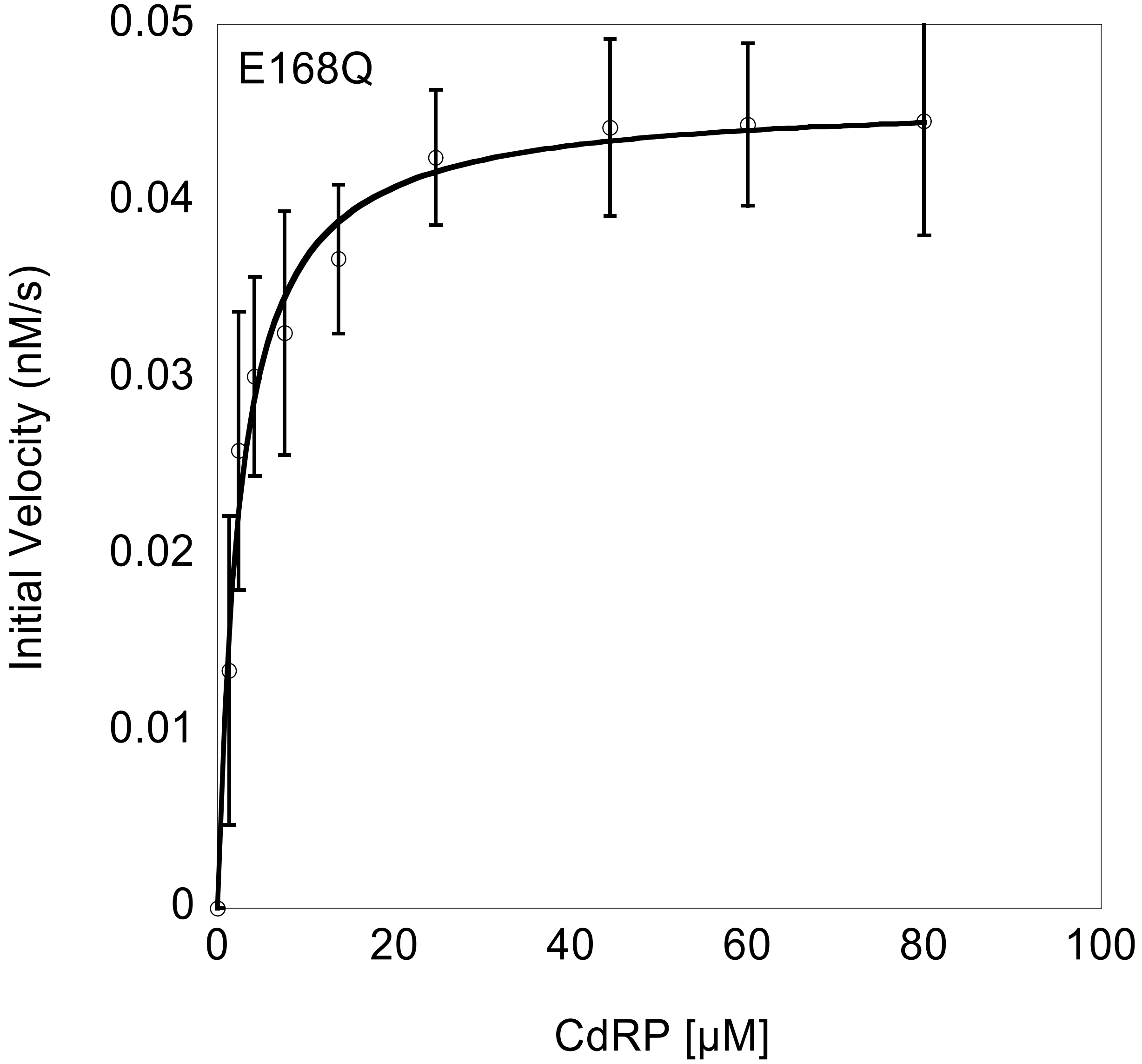

Fig. S9. Plot of Glu168Gln *Mt*IGPS velocity in nM/s graphed against CdRP concentration (μM) obtained at pH of 7.5 and 20 μM enzyme concentration. Each data point represents an average of three measurements with SEs and the data were fitted to the Michaelis-Menten equation with k_{cat} of 2.3 x10^{-6} ± 0.1x10^{-6} s^{-1} and K_M of 2.4 ± 0.3 μM.

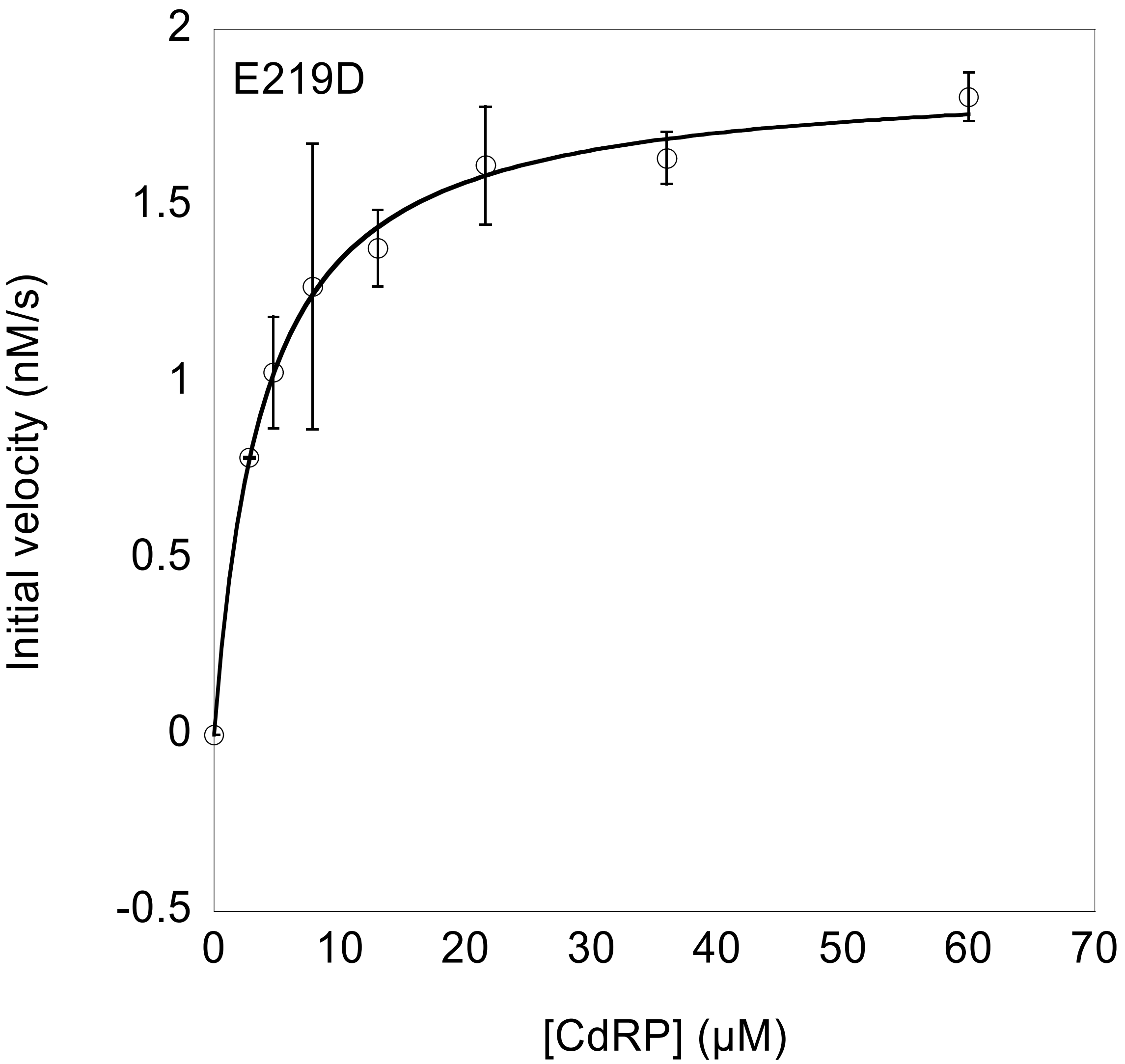

Fig. S10. Plot of Glu219Asp *Mt*IGPS velocities in nM/s graphed against CdRP concentration in µM obtained at pH of 7.5 and 15 µM enzyme concentration. Each data point represents an average of four measurements with SEs and the data were fitted to the Michaelis-Menten equation to obtain a k_{cat} of $2.70 \times 10^{-4} \pm 0.02 \times 10^{-4}$ s^{-1} and a K_M of 3.9 ± 0.3 µM.

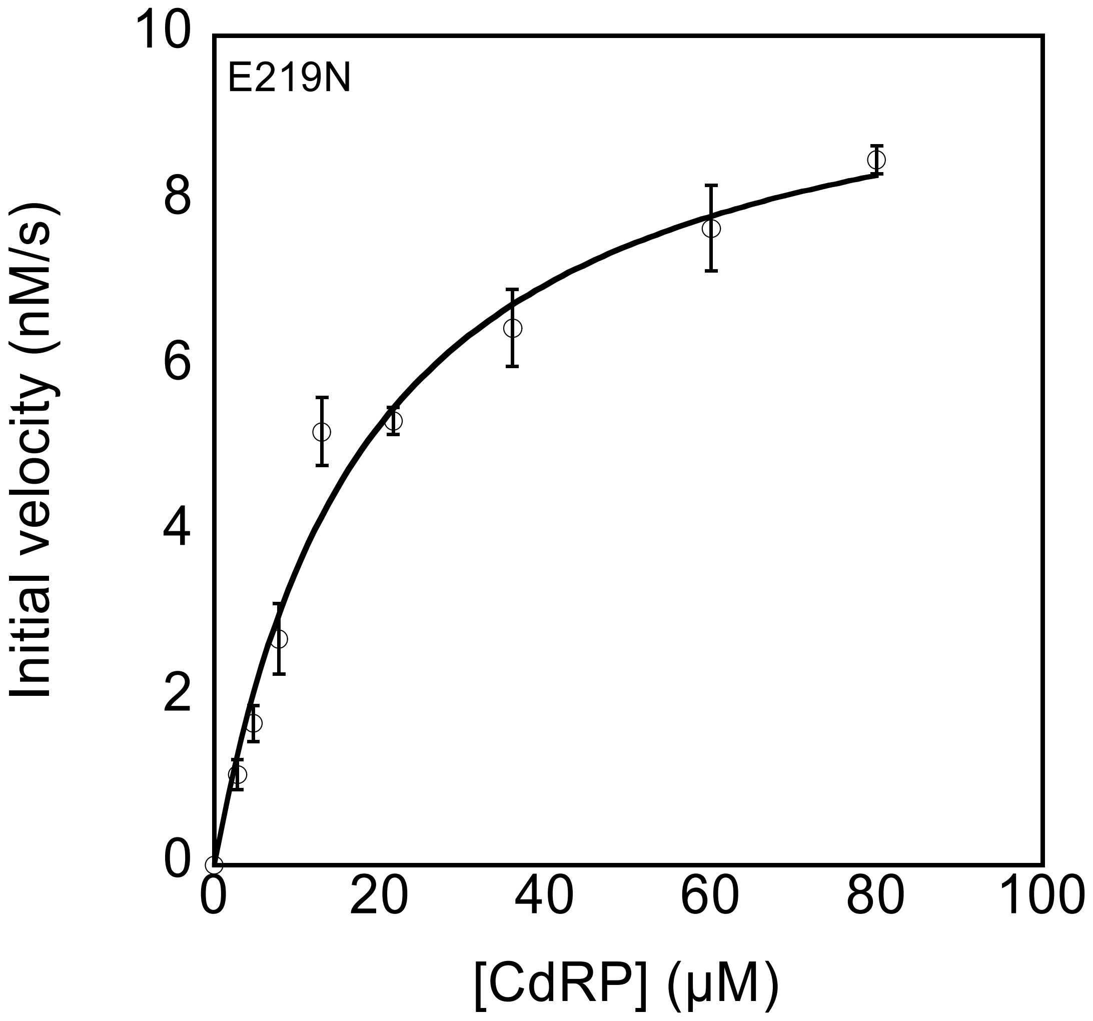

Fig. S11. Plot of Glu219Asn *Mt*IGPS velocities in nM/s graphed against CdRP concentration in µM obtained at pH of 7.5 and 2 µM enzyme concentration. Each data point represents an average of three measurements with SEs and the data were fitted to the Michaelis-Menten equation to obtain a k_{cat} of 0.0103 ± 0.0007s^{-1} and a K_M of 18 ± 3 µM.

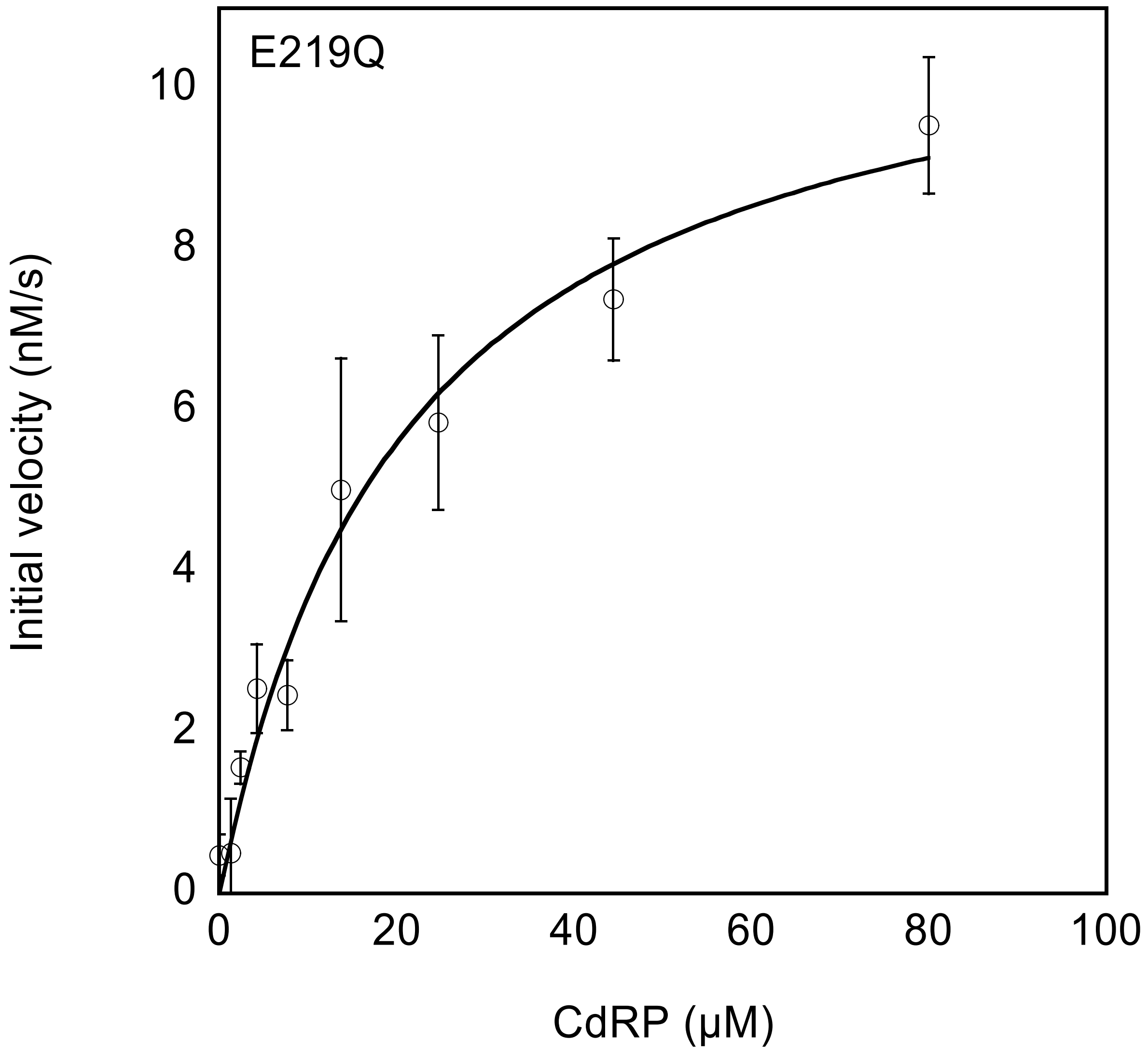

Fig. S12. Plot of Glu219Gln *Mt*IGPS velocities in nM/s graphed against CdRP concentration in μM obtained at pH of 7.5 and 0.05 μM enzyme concentration. Each data point represents an average of three measurements with SEs and the data were fitted to the Michaelis-Menten equation to obtain a k_{cat} of 0.23 ± 0.02 s^{-1} and a K_M of 21 ± 4 μM.

```
                  E57 K59
M. tuberculosis     MSPATVLDSILEGVRADVAAREASVSLSEIKAAAAAAPPPLDVMAA----LREPGIGVIA 56
E. coli             --MQTVLAKIVADKAIWVEARKQQQPLASFQN--EVQPSTRHFYDA----LQGARTAFIL 52
P. aeruginosa       MSVPTVLQKILARKAEEVAERRARVNLAEVERLARSADAPRGFANALLERAKRKEPAVIA 60
B. subtilis         -----MLEKIIKQKKEEVKTLVLPVE---------QPFEKRSFKEALA--SPNRFIGLIA 44
T. maritima         ---MRRLWEIVEAKKKDILEIDG-------ENLI-VQRRNHRFLEVL---SGKERVKIIA 46
S. solfataricus     --MPRYLKGWL----KDVVQLSLRRP----SFRASRQRPIISLNERILEFNKRNITAIIA 50
                          *   :      :                        .              .*

M. tuberculosis     EVKRASPSAGALATIADPAKLAQAYQDGGARIVSVVTEQRRFQGSLDDLDAVRASVSIPV 116
E. coli             ECKKASPSKGVIRDDFDPARIAAIYKHY-ASAISVLTDEKYFQGSFNFLPIVSQIAPQPI 111
P. aeruginosa       EIKKASPSKGVLREHFVPAEIARSYEAGGAACLSVLTDVDFFQGADAYLKEARAACALPV 120
B. subtilis         EVKKASPSKGLIKEDFVPVQIAKDYEAAKADAISVLTDTPFFQGENSYLSDVKRAVSIPV 104
T. maritima         EFKKASPSAGDINADASLEDFIRMYDEL-ADAISILTEKHYFKGDPAFVRAARNLTCRPI 105
S. solfataricus     EYKRKSPSGLDVE--RDPIEYSKFMERY-AVGLSILTEEKYFNGSYETLRKIASSVSIPI 107
                    * *: ***   :             .   *  :*::*:   *:*    :         *:
                    K119                                             E168
M. tuberculosis     LRKDFVVQPYQIHEARAHGADMLLLIVAALEQSVLVSMLDRTESLGMTALVEVHTEQEAD 176
E. coli             LCKDFIIDPYQIYLARYYQADACLLMLSVLDDDQYRQLAAVAHSLEMGVLTEVSNEEEQE 171
P. aeruginosa       VRKDFMIDPYQIVEARAIGADCILLIVSALDDVLMAELAATAKSVGLDVLVEVHDGTELE 180
B. subtilis         LRKDFI-DSLQVEESRRIGADAILLIGEVLDPLHLHELYLEAGEKGMDVLVEVHDASTLE 163
T. maritima         LAKDFYIDTVQVKLASSVGADAILIIARILTAEQIKEIYEAAEELGMDSLVEVHSREDLE 165
S. solfataricus     LMKDFIVKESQIDDAYNLGADTVLLIVKILTERELESLLEYARSYGMEPLIEINDENDLD 167
                    : ***  .  *:  :    **  *::   *      .:   : .  :  * *:      :
                                                              E219
M. tuberculosis     RALKA-GAKVIGVNARDLMTLDVDRDCFARIAPGLPSSVIRIAESGVRGTADLLAYAGAG 235
E. coli             RAIAL-GAKVVGINNRDLRDLSIDLNRTRELAPKLGHNVTVISESGINTYAQVREL-SHF 229
P. aeruginosa       RALKTLDTPLVGINNRNLHTFEVSLETTLDLLPEIPRDRLVVTESGILNRADVELMEVSE 240
B. subtilis         QILKVFTPDILGVNNRNLKTFETSVKQTEQIASLVPKESLLVSESGIGSLEHLTFVNEHG 223
T. maritima         KVFSVIRPKIIGINTRDLDTFEIKKNVLWELLPLVPDDTVVVAESGIKDPRELKDL-RGK 224
S. solfataricus     IALRI-GARFIGINSRDLETLEINKENQRKLISMIPSNVVKVAESGISERNEIEELRKLG 226
                      :      .:*:* *:*  :. . .    :   :  .   ::***:    .:

M. tuberculosis     ADAVLVGEGLVTSGDPRAAVADLVTAGTHPSC--PKPAR- 272
E. coli             ANGFLIGSALMAHDDLHAAVRRVLLGENKVCGLTRGQDAK 269
P. aeruginosa       VYAFLVGEAFMRADDPGLELKRLFFQERGGVVLGADPD-- 278
B. subtilis         ARAVLIGESLMRQTSQRKAIHALFRE-------------- 249
T. maritima         VNAVLVGTSIMKAENPRRFLEEMRAWSE------------ 252
S. solfataricus     VNAFLIGSSLMRNPEKIKEFIL------------------ 248
                    . ..*:* .::   .    .
```

Fig. S13. Amino acid sequence alignment for *Mt*IGPS and five orthologs. *Mt*IGPS residues Glu57, K59, Lys119, Glu168, and Glu219 are labeled and their alignment positions are highlighted in bold. The asterisk (*), colon (:), and period (.) indicate that a residue is fully conserved, similar, and weakly similar, respectively.

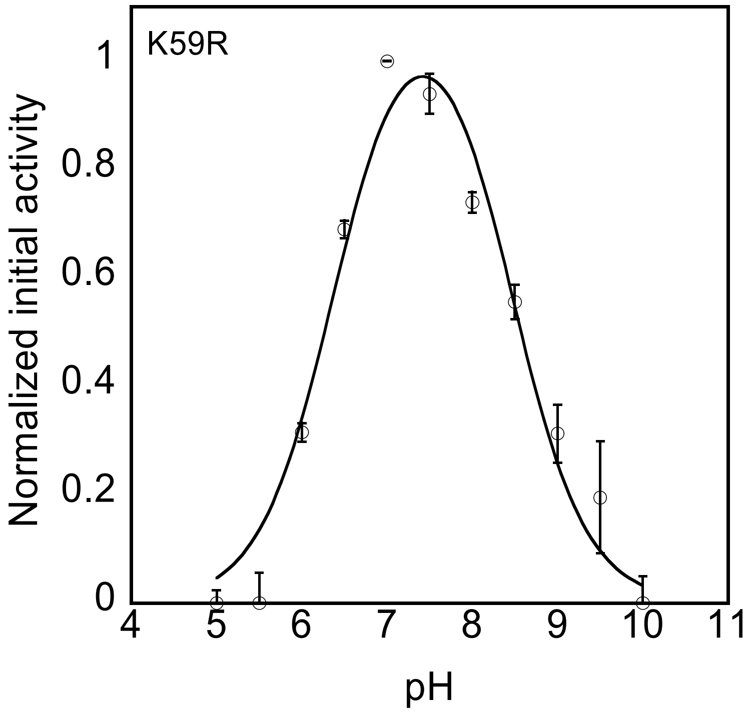

Fig. S14. Plot of Lys59Arg *Mt*IGPS normalized initial velocities vs. pH values obtained at 25 °C and 60 μM enzyme. Each data point is an average of three measurements ± SE. The initial velocities are expected to be governed by k_{cat} because the CdRP concentration was maintained above K_M. The pKa1 and pKa2 values, associated with the ascending and descending limbs of the rate-pH profile, were found to be 6.4 ± 0.1 and 8.5 ± 0.1 (SE), respectively.

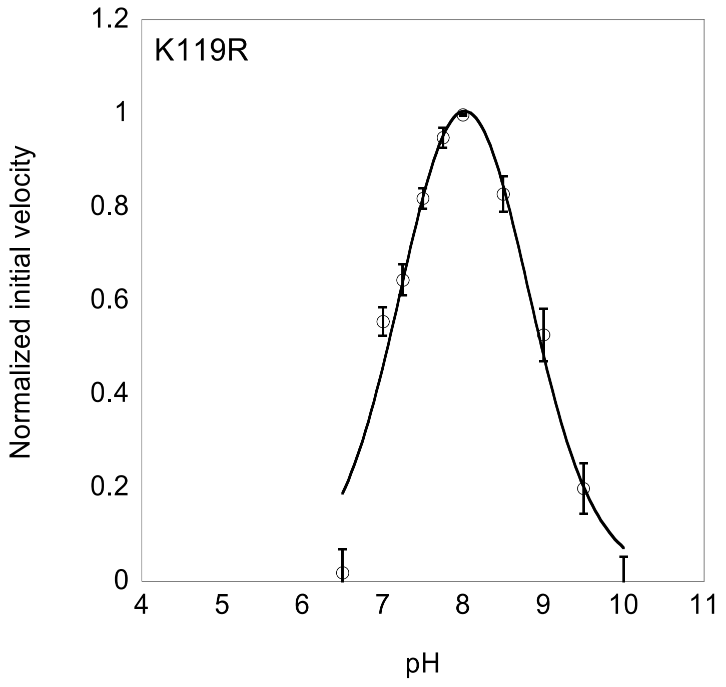

Fig. S15. Plot of Lys119Arg *Mt*IGPS normalized initial velocities vs. pH values obtained at 25 °C and 60 μM enzyme. The initial velocities are expected to be governed by k_{cat} because the CdRP concentration was maintained above K_M. Each data point is an average of seven measurements ± SE. The pKa1 and pKa2 values, associated with the ascending and descending limbs of the rate-pH profile, were found to be 7.3 ± 0.1 and 8.7 ± 0.1 (SE), respectively.

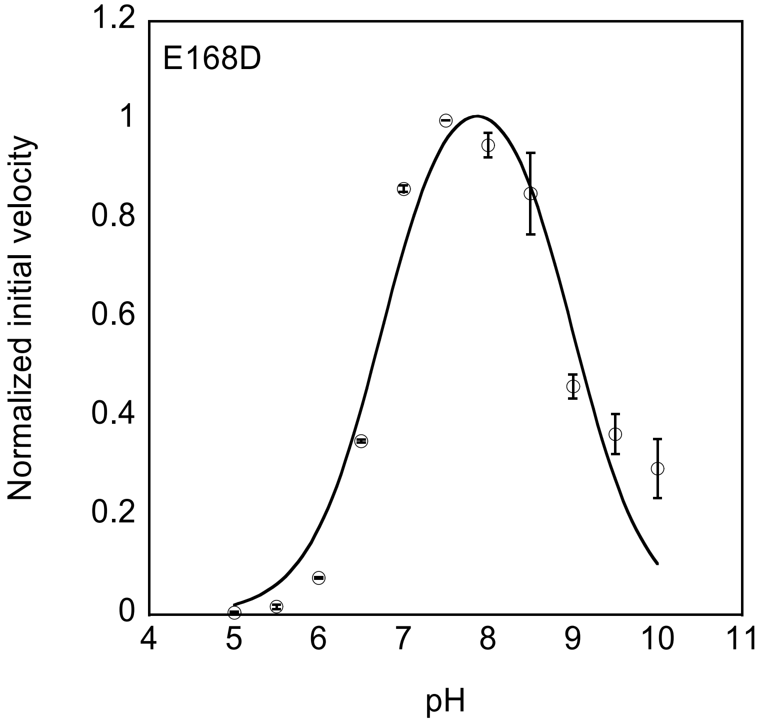

Fig. S16. Plot of Glu168Asp *Mt*IGPS normalized initial velocities (±SE) vs pH values obtained at 25 °C and at 1 μM enzyme (n=4). The initial velocities are expected to be governed by k_{cat} because the CdRP concentration was maintained above K_M. The pKa1 and pKa2 values, associated with the ascending and descending limbs of the rate-pH profile, were found to be 6.8 ± 0.2. and 9.0 ± 0.2 (SE), respectively.

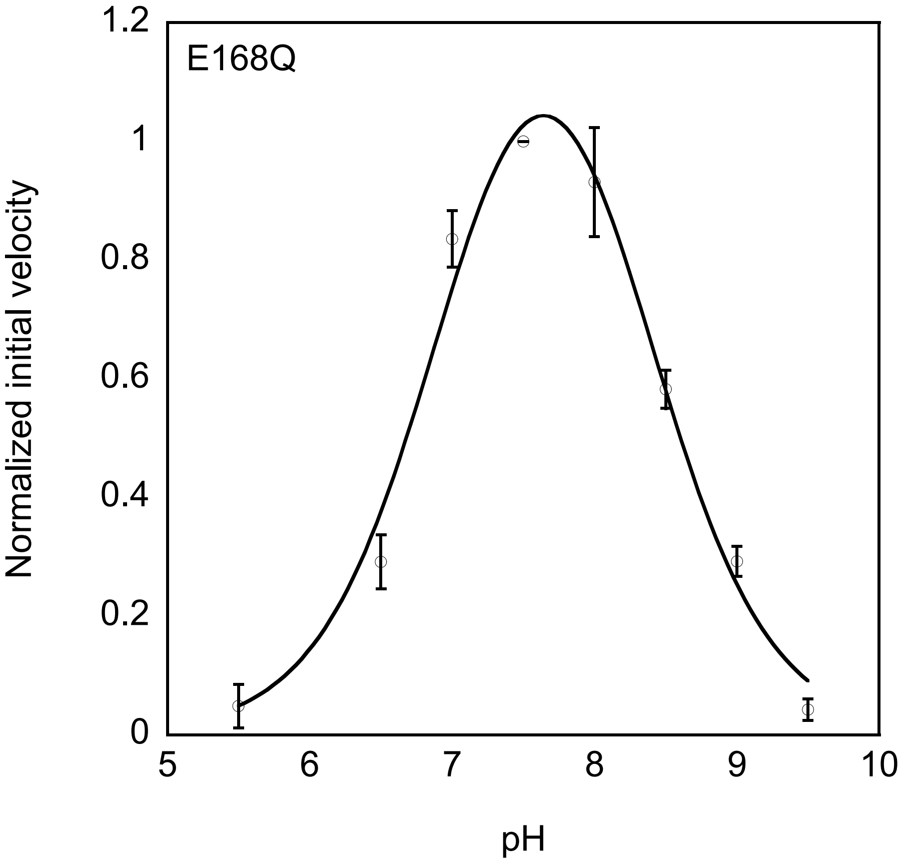

Fig. S17. Plot of Glu168Gln *Mt*IGPS normalized initial velocities (±SE) vs pH values obtained at 25 °C and at 20 μM enzyme (n=2). The initial velocities are expected to be governed by k_{cat} because the CdRP concentration was maintained above K_M. The pKa1 and pKa2 values, associated with the ascending and descending limbs of the rate-pH profile, were found to be 7.0 ± 0.1 and 8.3 ± 0.1 (SE), respectively.
